# ERK1/2 mitogen‐activated protein kinase dimerization is essential for the regulation of cell motility

**DOI:** 10.1002/1878-0261.13732

**Published:** 2024-09-12

**Authors:** Dalia de la Fuente‐Vivas, Vincenzo Cappitelli, Rocío García‐Gómez, Sara Valero‐Díaz, Camilla Amato, Javier Rodriguéz, Santiago Duro‐Sánchez, Alexander von Kriegsheim, Michael Grusch, José Lozano, Joaquín Arribas, Berta Casar, Piero Crespo

**Affiliations:** ^1^ Instituto de Biomedicina y Biotecnología de Cantabria (IBBTEC) Consejo Superior de Investigaciones Científicas (CSIC) – Universidad de Cantabria Santander Spain; ^2^ Centro de Investigación Biomédica en Red de Cáncer (CIBERONC) Instituto de Salud Carlos III Madrid Spain; ^3^ Cancer Research UK Scotland Centre, Institute of Genetics and Cancer University of Edinburgh UK; ^4^ Cancer Research Program Hospital del Mar Medical Research Institute (IMIM) Barcelona Spain; ^5^ Department of Biochemistry and Molecular Biology Universitat Autónoma de Barcelona Spain; ^6^ Preclinical and Translational Research Program Vall d'Hebron Institute of Oncology (VHIO) Barcelona Spain; ^7^ Center for Cancer Research Medical University of Vienna Austria; ^8^ Universidad de Málaga and Instituto de Investigación Biomédica de Málaga y Plataforma en Nanomedicina – IBIMA, Plataforma Bionand Spain; ^9^ Present address: Universidad de Burgos Burgos Spain

**Keywords:** cell motility, ERK, KSR, MAP kinases, scaffold proteins

## Abstract

ERK1/2 mitogen‐activated protein kinases (ERK) are key regulators of basic cellular processes, including proliferation, survival, and migration. Upon phosphorylation, ERK becomes activated and a portion of it dimerizes. The importance of ERK activation in specific cellular events is generally well documented, but the role played by dimerization is largely unknown. Here, we demonstrate that impeding ERK dimerization precludes cellular movement by interfering with the molecular machinery that executes the rearrangements of the actin cytoskeleton. We also show that a constitutively dimeric ERK mutant can drive cell motility *per se*, demonstrating that ERK dimerization is both necessary and sufficient for inducing cellular migration. Importantly, we unveil that the scaffold protein kinase suppressor of Ras 1 (KSR1) is a critical element for endowing external agonists, acting through tyrosine kinase receptors, with the capacity to induce ERK dimerization and, subsequently, to unleash cellular motion. In agreement, clinical data disclose that high *KSR1* expression levels correlate with greater metastatic potential and adverse evolution of mammary tumors. Overall, our results portray both ERK dimerization and KSR1 as essential factors for the regulation of cell motility and mammary tumor dissemination.

## Graphical abstract

In mammary tumor cells, ERK dimerization is both necessary and sufficient for inducing cellular migration. The scaffold protein KSR1 is a critical element for endowing external agonists, acting through tyrosine kinase receptors, with the capacity to induce ERK dimerization and, subsequently, unleashing cellular motion, as a consequence of which tumor cell migration and metastatic dissemination are promoted.

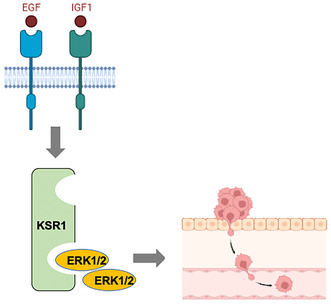

**Abbreviations**
EGF: epidermal growth factor
ELK: Ets‐like kinase
ERK: extracellular signal‐regulated kinase
FAK: focal adhesion kinase
IGF: insulin‐like growth factor
KSR: kinase suppressor of Ras
MEK: MAP kinase ERK kinase
MLC: myosin light chain
MLCK: myosin light chain kinase
MYPT: myosin phosphatase regulatory subunit
PAGE: protein acrylamide gel electrophoresis
RAS: rat sarcoma
RSK: p90 ribosomal S6 kinase
RTK: receptor tyrosine kinase
siRNA: small interfering RNA

## 1 Introduction

Extracellular signal‐Regulated Kinase 1 and 2 Mitogen‐Activated Protein Kinases (ERK, hereafter) are indispensable mediators in the transduction of external stimuli‐evoked signals to the interior of the cell, unleashing biochemical processes and genetic programs fundamental for the regulation of basic cellular outcomes such as proliferation, differentiation, survival, and motility, plus many other cell type‐specific cellular events essential for maintaining organismal homeostasis. As such, unregulated signal flux through the RAS–ERK axis underlies multiple pathological conditions, including cancer [1, 2, 3].

A fraction of the ERK pool is known to dimerize in response to its phosphorylation [4]. It has been proposed that dimerization affects ERK activity levels [5]. In addition, dimerization critically impacts on the spatial regulation of ERK activity [6] by specifically orchestrating ERK extranuclear but not nuclear signaling [7, 8]. This is attained through the participation of Scaffold Proteins, such as KSR1 (Kinase Suppressor of Ras) [9]. This was the first mammalian scaffold described as a protein binding to C/BRAF, MEK1/2 and ERK1/2 [9]. KSR1 is a ubiquitous, multidomain, cytoplasmic protein that rapidly translocates to the plasma‐membrane upon activation to regulate RAS–ERK signals [10, 11]. KSR1, like other scaffold proteins, provides spatial selectivity to ERK signals, by regulating their activity in a sublocalization‐specific fashion [2, 12, 13, 14, 15]. In this respect, Scaffold Proteins play an essential role in the process of ERK dimerization, acting as platforms where ERK dimers are assembled in the cytoplasm [7, 10, 16, 17]. Accordingly, genetic ablation of scaffolds like KSR1 and others markedly impairs RAS–ERK signaling, thereby thwarting RAS‐driven tumorigenesis [18, 19, 20]. Likewise, impeding ERK dimerization using small inhibitory molecules also forestalls neoplastic progression of tumor cells harboring RAS–ERK pathway oncogenes [17, 21]. However, detailed knowledge about the role that dimerization plays on specific ERK‐regulated cellular events, both physiological and at the heart of carcinogenesis, is largely missing.

Cell motility is essential for many physiological processes like embryogenesis, development, inflammation, and wound healing, among others. It also plays a central role in pathological conditions such as cancer, underlying local invasion, intra and extravasation and colonization of distant organs by tumor cells. Cellular movement entails coordinated, spatio‐temporally defined events, including the initial formation of protrusions at the cell leading edge; the formation and disappearance of cell‐substrate adhesions; and the final retraction of the cellular tail [22]. ERK activation plays an important role in the regulation of these processes, via phosphorylation of key components of the molecular machinery that makes cellular movement happen. Consistently, disruption of ERK signaling, either by genetic mutations or pharmacological inhibition, prevents motility in many cell types [1, 23, 24]. Yet, as of today, whether ERK monomer/dimer status impacts on its ability for regulating cell motility is completely unknown.

Breast cancer is the most common malignancy among women. It is well known that the RAS–ERK pathway plays an important role in the onset, evolution and dissemination of mammary tumors. Both as an effector pathway for the epidermal growth factor receptor family and also as an upstream regulator of the estrogen receptor, two critical participants in breast neoplasia [25]. Primary tumors of ‘node‐positive’ patients display higher ERK activity than those from ‘node‐negative’ patients and survival analyses show that low ERK activity in primary tumors is associated to relapse‐free survival of patients [26], suggesting a positive correlation between ERK activity and the metastatic potential of breast tumor cells. But the extent to which such ERK activity can be associated to its specific functions in monomeric or dimeric form is absolutely unknown. In this study, we present evidence demonstrating that ERK dimerization is both necessary and sufficient for inducing cellular movement in mammary tumor cells. Unveiling ERK dimers as promising targets for preventing the metastatic progression in breast cancer.

## 2 Materials and methods

All data shown are representative of at least three independent experiments.

### 2.1 Cell lines, drugs and reagents

Cell lines were grown in a humidified incubator at 37 °C and 5% CO_2_ in Dulbecco's modified eagle medium (DMEM) (Thermo Fisher, Waltham, MA, USA) supplemented with 10% Fetal Bovine Serum (Gibco, Grand Island, NY, USA) and 1% Penicillin‐Streptomycin (10 000 U·mL^−1^) (Thermo Fisher). MCF‐7 (RRID:CVCL_0031); MDA‐MB‐231 (RRID:CVCL_0062); HepG2 (RRID:CVCL_0027) and Caco2 (RRID:CVCL_0025) cells were obtained from ATCC (Manassas, VA, USA). The identity of the cell lines was verified by Short Tandem Repeat (STR) authentication. STR identity profile was analyzed using multiplex PCR assays using the Promega PowerPlex 18D system and the ThermoFisher Scientific genemapper ID‐X v1.2 software for analysis of the amplicons. EGF (epidermal growth factor) was from Sigma‐Aldrich (USA; #E9644, Madrid, Spain); IGF (insulin‐like growth factor‐1) was from Peprotech (#100‐11, Cranbury, NJ, USA). DEL 22379 was synthesized at Vichem Chemie (BUdapest, Hungary). U0126 was from Promega (#V1121, Madrid, Spain). All experiments were performed using mycoplasma free cells (Fig. S1). LookOut Mycoplasma qPCR Detection Kit (Cat. no. MP0040; Sigma‐Aldrich) was used following manufacturer instructions.

### 2.2 Gene knockdown and overexpression

siRNA against KSR1 (#sc‐35762) and control si RNA (control #sc‐37007) was purchased from Santa Cruz Biotechnology (Heidelberg, Germany). siRNAs were transfected with Lipofectamine™ RNAiMAX Transfection Reagent (#13778150; Thermo Fisher Scientific) following manufacturer's directions. MCF‐7 and MDA‐MB231 cells were transfected using Lipofectamine 3000 (#L3000015; Invitrogen, Thermo Fisher, Whatham, MA, USA) as specified by the manufacturers.

### 2.3 Western blot analyses

Cell plates were collected on ice, cells were washed in cold 1× PBS and harvested in 200–500 μL of lysis buffer (20 mm HEPES pH 7.5, 10 mm EGTA, 40 mm β‐Glycerophosphate, 1% NP40, 2.5 mm MgCl_2_, 1 mm NaVO_4_, 1 mm DTT and protease inhibitors: 10 μg·mL^−1^ of aprotinin and 10 μg·mL^−1^ of leupeptin). Cell lysates were cleared at 2700 *g* for 10 min at 4 °C and protein concentration was quantified using the Bradford Method at 620 nm. 5× laemmli loading buffer was added to samples of 30 μg protein and the mix was boiled at 95 °C for 5 min. Proteins were resolved by sodium dodecyl sulfate (SDS)/poliacrylamide gel electrophoresis (PAGE). Native gel protein electrophoresis was performed as described [17]. Gels were transferred to Nitrocellulose membranes (AmershamProtran Supported 0.45 NC; GE Healthcare Life Sciences, Chicago, IL, USA). Membranes were blocked in Tris‐Buffered Saline‐Tween (TBS‐T) containing 4% BSA (blocking solution). Blots were incubated from 1 h at room temperature to O/N at 4 °C (depending on the antibody performance) with the different antibodies prepared in blocking solution. Subsequently, the blots were incubated for 1 h shaking at room temperature with anti‐rabbit Immunoglobulin (Ig) (#170‐5046; Bio‐Rad, Madrid, Spain) or anti‐mouse Ig (#170‐5047; Bio‐Rad) secondary antibodies conjugated with peroxidase (1 : 10 000) in 4% milk (GE Healthcare) TBS‐T. Proteins were detected by chemiluminescence with an enhanced chemiluminescent system (ECL) and signals were visualized and recorded with ChemiDoc MP Imaging System (Bio‐Rad).

### 2.4 Co‐immunoprecipitation assays

Cell lysates were centrifuged at 13 000 r.p.m./4 °C/10 min. The cleared lysates were quantified and 30 μg of protein were separated and 5× loading buffer was added to be used as total lysate. 0.5–1 μg of the antibody specific for immunoprecipitation was added to 300 μg of protein and incubated rocking at 4 °C/2 h up to O/N. 20 μL of protein G‐Sepharose 4B (#17‐0756‐01; GE Healthcare) were added and incubated 20 min/4 °C shaking. The immunocomplexes were precipitated by centrifugation. Beads were washed once with lysis buffer and twice with cold 1× PBS; 1% NP‐40. Beads were resuspended in 20 μL of 2.5× loading buffer Laemmli and boiled for 5 min, then analyzed by SDS/PAGE as previously described. Co‐immunoprecipitations were repeated at least three times in independent experiments.

### 2.5 Antibodies utilized

Mouse monoclonal anti‐Flag M2 (Sigma‐Aldrich) Cat# F1804, RRID:AB_262044; Mouse monoclonal anti‐p‐ERK (E‐4) (Santa Cruz) Cat# sc‐7383, RRID:AB_627545; Mouse monoclonal anti‐MAP Kinase, diphosphorylated ERK‐1&2 (Sigma‐Aldrich) Cat# M9692, RRID:AB_260729; Mouse monoclonal anti‐MAP Kinase, diphosphorylated ERK‐1&2 (Sigma‐Aldrich) Cat# M8159, RRID:AB_477245; Mouse monoclonal anti‐ERK1&2 (Santa Cruz) Cat# sc‐514302, RRID:AB_2571739; Rabbit anti‐KSR1 [EPR2421Y] (Abcam, Cambridge, UK) Cat# ab68483, RRID:AB_11157290; Rabbit Anti‐MYL12 (phosphoS19) (Abcam) Cat# AB2480, RRID:AB_303094; Rabbit Anti‐Myosin Light Chain 2 (Cell Signaling, Danvers, MA, USA) Cat# 3672, RRID:AB_10692513; Rabbit anti‐Phospho‐MYLK (Ser1760) (Thermo Fisher) Cat# 44‐1085, RRID:AB_2533570; Anti‐FLAG tag. Mouse monoclonal (Sigma‐Aldrich) Cat# F1804, RRID:AB_262044; Anti‐HA tag. Mouse monoclonal (F‐7) (Santa Cruz) Cat# sc‐7392, RRID:AB_627809; Rabbit anti‐Phospho‐Paxillin (Invitrogen) Cat# 44‐720G, Mouse Monoclonal anti‐Paxillin (BD Bioscience, Barcelona, Spain) Cat# 610051, RRID:AB_397463; Mouse monoclonal Anti‐phospho‐Rsk1 (Thr359/Ser363) (Sigma‐Aldrich) Cat# ABS1849, RRID:AB_2181932; Rabbit Anti‐Rsk (Santa Cruz) Cat# sc‐74575, RRID:AB_2181931; Goat anti‐ rabbit IgG (H + L) HRP Conjugate (Bio‐Rad) Cat# 1706515, RRID:AB_11125142; Goat anti‐Mouse IgG (H + L) HRP Conjugate (Bio‐Rad) Cat# 1706516, RRID:AB_2921252; Rabbit anti‐GAPDH (0411) (Santa Cruz) Cat# sc‐47724, RRID:AB_627678; Texas Red X Phalloidin (Invitrogen) Cat# 7471.

### 2.6 Proliferation assays

Proliferation assays were performed as described [27], using the PrestoBlue Cell Viability Reagent (#A13261; Thermo Fisher). Changes in metabolic activity and, indirectly, cell number can be detected by a media color change that can be measure using absorbance‐based plate readers, using 600 nm as a reference wavelength and monitoring reagent absorbance at 570 nm. The cells were counted by Neubabuer chamber or Nucleocounter (method based on propidium iodide staining). 6000 cells were plated per well in three 96‐well plates, one for each time point (24, 48, and 72 h) and three replicates per condition. At the programmed times, 10 μL of room temperature PrestoBlue Reagent was added and incubated in the dark at 37 °C and the absorbance monitored.

### 2.7 Liquid chromatography‐mass spectrometry (LC–MS/MS) analysis

Performed as described [28], in MCF7 cells transfected with HA‐ERK2 (1 μg). The EGF and IGF treatments were done 24 h post‐transfection. The cells were lysed in lysis buffer (1% Triton X‐100, 150 mm NaCl, 20 mm Tris–HCl pH 7.5, 1 mm EDTA pH 7.5). Immunoprecipitation, washing, and digestion were performed on a KingFisher Duo robotic station (Thermo Fisher Scientific). 5 μL of magnetic antibody bead slurry, anti‐HA beads (MBL Bio, Schaumburg, IL, USA), was diluted in 100 μL of lysis buffer and loaded in row H of a 96 deep‐well plate. 500 μL of lysate was loaded into row G, 300 μL of lysis buffer were loaded into rows E and F. 300 μL of Wash buffer (150 mm NaCl, 20 mm Tris–HCl pH 7.5, 1 mm EDTA pH 7.5) were loaded into rows B–D. Row A contained the 100 μL of digest buffer (2 m Urea, 50 mm Tris–HCl pH 7.5, 1 mm DTT, 5 μg·mL^−1^ porcine trypsin (Promega) 5 μg·mL^−1^ GluC (Promega)). The robot picked up beads in row H, transported them to row G and released and mixed them for 2 h. Beads were picked up and released subsequently into rows F to B with 1 min. mixing in between. The washed beads were then transported into row A and digested at 27 °C for 30 min. under mixing. Beads were then removed and digested for 8 h at 37 °C. After iodoacetamide modification and acidification of the samples, the peptide mixtures were desalted using homemade C18 tips. The desalted and lyophilized peptides were resuspended in 0.1% TFA and subjected to mass spectrometric analysis by reversed‐phase nano‐LC–MS/MS. Mass spectrometry: 5 μL of the resuspended peptides were analyzed by reversed‐phase nano‐LC–MS/MS using a nano‐Ultimate 3000 liquid chromatography system and a QExactive plus or Lumos Fusion mass spectrometer (both Thermo Fisher Scientific). Flow rates were 400 nL·min^−1^. Peptides were loaded onto a self‐packed analytical column (μChrom 1.6, 0.075 mm × 25 cm) using a 67‐min gradient Buffer A, 2% acetonitrile 0.5% acetic acid, Buffer B, 80% acetonitrile, 0.5% acetic acid; (0–16 min: 2% buffer B, 16–56 min: 3–35% buffer B, 56–62 min: 99% buffer B; 62–67 min 2% buffer B). The QExactive was operated in top‐12, data‐dependent mode with a 30‐s dynamic exclusion range. Full‐scan spectra recording in the Orbitrap was in the range of *m/z* 350 to *m/z* 1650 (resolution: 70 000; AGC: 3e6 ions). MS2 was performed with an isolation window of 1.4, AGC 5e4, HCD collision energy of 26, Scan range from 140 200 ms maximum injection time. The Lumos was operated in data‐dependent mode with a 10‐s dynamic exclusion range. Full‐scan spectra recording in the Orbitrap was in the range of *m/z* 350 to *m/z* 1400 (resolution: 240 000; AGC: 7.5e5 ions). MS2 was performed in the ion trap, isolation window 0.7, AGC 2e4, HCD collision energy of 28, rapid scan rate, Scan range 145–1450 *m/z*, 50 ms maximum injection time and an overall cycle time of 1 s.

#### 2.7.1 Database search

The mass spectrometry raw data were analyzed by the maxquant and andromeda software package [29] using the pre‐selected conditions for analysis (specific proteases, 2 missed cleavages, 7 amino acids minimum length). Protease was set to trypsin. Carbamylation (C) was selected as fixed modification. Variable modifications were N‐terminal acetylation (protein), oxidation (M). FDR was set to 0.01. MS/MS spectra were searched against the human Uniprot database and the maxquant contaminant database with a mass accuracy of 4.5 p.p.m. (for MS) and 20 p.p.m. or 0.5 Da (MS/MS OT or IT). Peak matching was selected and was limited to within a 0.7 min. elution window with a mass accuracy of 4.5 p.p.m.

### 2.8 Cell migration chemotaxis

Cell migration chemotaxis was examined in Transwell cell culture chambers with 8 μm pore filters (Corning, NY, USA). 0.1 × 10^6^ cells·mL^−1^ in 200 μL of serum‐free medium were added to the top of the Transwell and the chemoattractant was added to the bottom. Following 16 h incubation at 37 °C/5% CO_2_, the invading cells were fixed with 4% paraformaldehyde and stained with crystal violet −1% methanol, analyzed by microscopy and counted. Images were processed and analyzed using fiji image (NIH, Bethesda, MD, USA). Cell proliferation was monitored in parallel plates.

### 2.9 Cell migration assay over‐time

To measure cell migration in 2D, *in vitro* cultures of 1 × 10^5^ cells were seeded in a T6 plate and treated with drugs and stimuli. The cells grew for 48 h. Photos were taken by Microscopy Ti Eclipse FL invert new. Video microscopy was generated using a Nikon Visitron Live Cell System (Visitron Systems GmbH, Puchheim, Germany) with images taken every 30 min for 48 h. Migration of single cells were manually tracked using imagej to obtain coordinates for each cell and time points. For further analysis of migratory behavior including speed, mean squared displacement (MSD), directionality ratio (DR), and origin plots, the DiPer migration tool was used.

### 2.10 3D tumor spheroid invasion assay

4 × 10^4^ cells were resuspended in low viscosity medium (800 μL – 10% FBS medium and 200 μL of low viscosity methylcellulose; Sigma). 25 μL droplets of the medium were suspended on the lid of a 10 mm dish and cultured for 48 h at 37 °C/5% CO_2_. Spheroids were resuspended gently in 5 mL of 1× PBS and centrifuged at 300 r.p.m./15 s. The spheroids were resuspended gently in collagen I solution (1.7 mg·mL^−1^ in 5× DMEM). 300 μL of this suspension were seeded in a 24‐well plate and incubated at 37 °C for 4 h until the matrix polymerized. Once the matrix polymerized, the 3D tumor spheroids the indicated treatments were initiated and growth was monitored after 48 and 72 h. Images were taken by optical microscopy at 4× magnification. To quantify the spheroid proliferation and invasion the imagej tool was used.

### 2.11 Immunofluorescence assay

Basically as described [30]. Cells were subsequently washed twice with cold 1× PBS for 5 min, followed by fixation with 4% paraformaldehyde for 10 min. The protocol was followed by two 1× PBS, one 0.1 m glycine, and two 1× PBS washes. Subsequently, cells were permeabilized for 5 min with 0.5% Triton X‐100 in 1× PBS and washed again with 1× PBS for 5 min. Cells were incubated with 0.05% Tween TM20 (Thermofisher) for 5 min to reduce surface tension. Diluted primary antibody was added as a drop (8 μL) over the glass and incubated for 1 h in a humidity chamber. Cells were washed twice for 5 min with 1× PBS and secondary antibody conjugated with a fluorophore was added for 1 h and then removed washing twice with 1× PBS. Finally, the glasses were set over a slide with mounting media with Prolong‐DAPI (Invitrogen) and sealed with clear nail polish. In the case of spheroids immunofluorescence assay, they were fixed for 10 min at room temperature in 20% paraformaldehyde/PBS. The cellular membranes were permeabilized and blocked with 0.3% Triton in 4% BSA/PBS for 20 min at room temperature. The primary antibody was diluted in 4% BSA/PBS and kept the primary antibody O/N at 4 °C. The next day, the spheroids were washed, and the secondary antibody was added and incubated for 2 h at RT. Finally, the spheroids were washed and DAPI was added in the second wash. Cells and spheroids were examined by fluorescence microscopy (photomicroscope Axiophot; Carl Zeiss, Madrid, Spain) or confocal microscopy (Leica TCS SP8, Hessen, Germany). The images were processed using imagej software.

### 2.12 Chick embryo model for spontaneous metastasis

Spontaneous metastasis in chick embryos was performed as described [17]. Briefly, MDA‐MB‐231 cells (2 × 10^5^) were grafted on the choryo‐allantoid membrane (CAM) of 10‐day embryos. Experiments were terminated on day 5 after grafting and primary tumors were excised and weighed. Portions of the CAM and liver were harvested and analyzed for the number of human cells by quantitative Alu PCR (Alu‐qPCR). The chick embryo CAM assay did not require administrative procedures for obtaining ethics committee approval for animal experimentation. The CAM was not innervated, and experiments were terminated before the development of centers in the brain associated with pain perception, making this a system not requiring animal experimentation permissions. All experiments were performed according to the national guidelines for animal care in accordance with the European Union Directives.

### 2.13 Patient‐derived xenograft tissue microarray of breast cancer patients

As previously described [31], tissue microarray (TMA) consisted of 51 patient‐derived xenograft (PDX) samples corresponding to newly diagnosed cancer patients operated at 12 de Octubre University Hospital (Madrid, Spain) and Virgen del Rocío Hospitals (Seville, Spain), between September 2019 and March 2022, prior to any treatment. All tissue was collected with informed, written consent from the patients under REB‐approved protocols at both institutions. Human breast tumors used in this study to establish PDXs were obtained following the institutional guidelines and approval of the institutional review boards at Vall d'Hebron University Hospital in accordance with the Declaration of Helsinki, license # VHIO (PR(AG)130/2015).

The patient samples and the associated data comply with all the guarantees of protection, security and confidentiality established in the applicable regulations (Law 14/2007 on Biomedical Research, Regulation (EU) 2016/679 of the European Parliament and of the Council of April 27, 2016) regarding the protection of natural persons and Organic Law 3/2018, of December 5, on the Protection of Personal Data and guarantee of digital rights (LOPDGDD), Law 41/2002, of November 14, basic regulation of patient autonomy and rights and obligations in terms of clinical information and documentation. The original studies were approved by the Institutional Review Board of Vall d'Hebron hospital.

For the PDX generation, fresh tumor samples were obtained from the Vall d'Hebron University Hospital following the institutional guidelines. Informed patient written consent, approved by the Ethics Committee for Clinical Research and Animal Research of Vall d'Hebron Hospital (PR(AG)130/2015), was obtained for the use of these samples. Regarding the patient cohort, material representative of the disease was obtained from Luminal HER positive; HER‐positive; and TNBC primary and metastatic breast cancer patients aged 18 years or older, that had received different treatments (doxorubicin; paclitaxel; trastuzumab; t‐dm1; lapatinib) at the Vall d'Hebron University Hospital. Tumor tissue was used to generate PDX and the corresponding TMAs. Mice were maintained and treated in accordance with the Facility Animal Care Committee at the Vall d'Hebron Institute of Oncology (VHIO). Excess breast tumor tissue was transported to the laboratory in ice‐cold transport medium (DMEM/F12, 50 μg·mL^−1^ gentamicin, 1× penicillin–streptomycin, 2.5 μg·mL^−1^ fungizone). Samples were cut into 1 mm fragments and transplanted into the fourth mammary fat pad of 5–7‐week‐old NOD.Cg‐PrkdcscidIl2rgtm1Sug/JicTac (NOG) (Janvier, RRID:IMSR_CRL:394) females under sterile conditions. Fine needle aspirates were washed in PBS, resuspended in PBS: matrigel (Corning) (1 : 1) and 50 μL was injected orthotopically. All the animal studies were performed in accordance with the ARRIVE (Animal research: Reporting of *in vivo* experiments) guidelines and the 3R rule of replacement, reduction and refinement principles. All the animals were housed with a goal of maximizing species‐specific behaviors and minimizing stress‐induced behaviors. Mice were treated in accordance with the Ethical Committee for the Use of Experimental Animals (IACUC) at the Vall d'Hebron Institute of Oncology (VHIO, Barcelona). Experimental animal License number 10303 approved by Generalitat de Catalunya (Spain).

Mice were palpated weekly and tumors measured using calipers and harvested for *in vivo* passaging when tumors reached endpoint (> 10 mm in the largest dimension). At the end of the experiment, after from 4 to 6 months, animals were euthanized using CO_2_ inhalation. Tumor growth was measured once per week and mice weights were recorded twice per week. If mouse welfare was compromised before tumor progression, tumors were harvested and implanted into another recipient mouse. In each passage, flash‐frozen and formalin‐fixed paraffin‐embedded (FFPE) samples were taken for genotyping and histological studies. Paraformaldehyde‐fixed and paraffin‐embedded blocks of PDX tissue were used to generate the corresponding TMAs by punching two 1‐mm spots of sample. Prior to staining, slides were deparaffinized and tissue was rehydrated. To allow the antibody to penetrate more easily to each cell, a permeabilization step was done, incubating specimens for 10 min with 0.1% IGEPAL (#I8896; Sigma‐Aldrich) diluted in 1× Tris‐Buffered Saline (TBS). Then, slides were washed twice with 1× TBS for 5 min, and non‐specific bindings were eliminated using a serum‐free blocking reagent, background punisher (BIOCARE Medical, Madrid, Spain) for 10 min. After that, 1.5% horse serum (HS) 1× PBS solution containing an anti‐rabbit KSR1 (1 : 100) antibody, or none of them (negative control), was incubated O/N at 4 °C in a humid chamber to avoid evaporation. Next day, slides were washed in 1× TBS (3 × 5 min) and endogenous peroxidase was quenched using 3× H_2_O_2_ diluted in 1× TBS for 20 min. Then, specimens were washed in 1× TBS (3 × 10 min) and incubated with an anti‐mouse/rabbit biotinylated secondary antibody (VECTOR Lab, Barcelona, Spain) diluted in 1% BSA 0.05% IGEPAL 1× TBS (1 : 500) for 1 h in a humid chamber. After that, slices were washed, as before, and specimens were incubated with Horseradish Peroxidase (HRP) Avidin D diluted in 1× TBS (1 : 500) for 30 min in a humid chamber. Slices were washed again and incubated with diaminobenzidine (DAB, #K3468; Dako, Santa Clara, CA, USA) for 5 min or until brownish color started to appear. Chromogenic reaction was stopped washing with tap water. To finish specimens were stained with Hematoxylin (#109253; Sigma‐Aldrich), dehydrated, cleared, and mounted with DPX (#06522; Sigma‐Aldrich). Images were taken at a Zeiss Axio Scope A1 microscope using 20×, or 40× objectives. Evaluation of KSR1expression was displayed as DAB signals, 8 field per slide by calculation of *H*‐score using color deconvolution TMA plugin fiji software.

### 2.14 Statistical analyses

Statistical analyses were performed using graphpad prism software (Boston, MA, USA). Each experiment was independently repeated at least three times. All values and error bars were represented as mean of the number of determinations with error bars representing ± SD. Two‐tailed unpaired *t* tests were used to determine statistical significance between two experimental groups as indicated in the respective figure legends where the number of independent experiments (*n*) is indicated.

## 3 Results

### 3.1 Inhibition of ERK dimerization impedes cellular movement

**Fig. 1:**
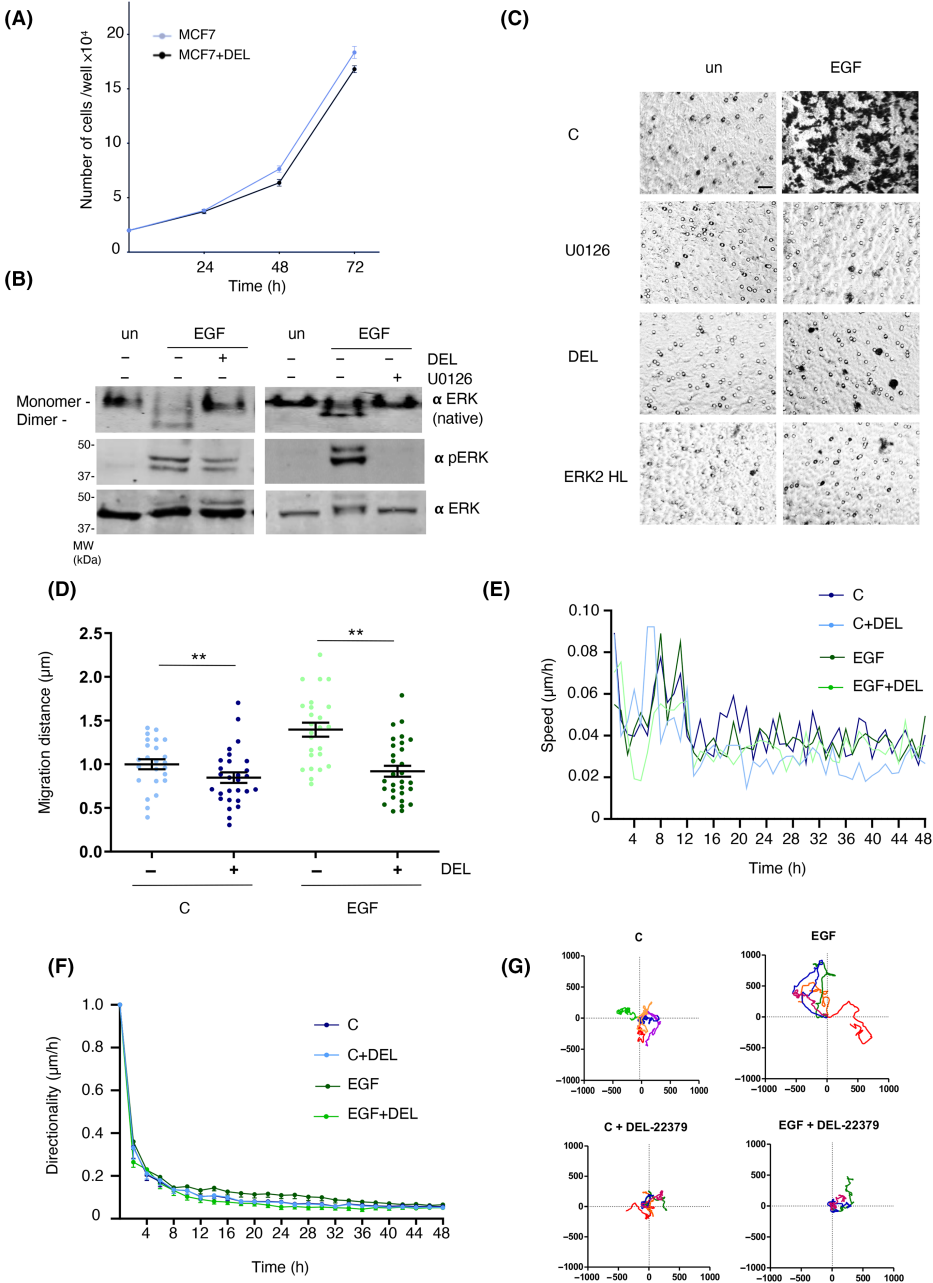
Inhibition of ERK dimerization prevents cell motility. (A) Effects of the ERK dimerization inhibitor on the proliferation of MCF7 cells. Where shown, cells were treated with DEL‐22379 (10 μm) for the indicated times. Data show average ± SEM from three independent experiments. (B) Effects of DEL‐22379 or U0126 (10 μm each) (+) on ERK dimerization and phosphorylation. Administered to 18 h‐starved MCF‐7 cells 30 min before treatment with EGF (50 ng·mL^−1^, 5 min) where indicated. un, unstimulated cells. Results are representative of three independent experiments. (C) Effects of ERK dimerization inhibition on cellular migration. Assayed in Transwell chambers (8 μm pore) using EGF (50 ng·mL^−1^, 48 h) as chemoattractant, in the presence of DEL‐223379, U0126 or previously transfected with the ERK2 HL dimerization‐inhibitory mutant (1 μg), where indicated. Results are representative of three independent experiments. Scale bar: 50 μm. (D–F) Effects of DEL‐22379 on the indicated parameters of cell motility in control (C) or EGF‐treated cells (50 ng·mL^−1^, 48 h), as indicated in each figure. Data show average ± SEM from three independent experiments. *P* values: **P < 0.01 by double‐tailed, unpaired Student *t*‐test. (G) Effects of ERK dimerization inhibition on individual cells motility, as determined by live cell video microscopy over 48 h, pictures taken every 30 min. Origin plots were calculated by DiPer.

In order to study the role of ERK dimerization in cellular motility of mammary tumor cells, we used the MCF7 epithelial tumor cell line. In these, it was found that treatment with the ERK dimerization inhibitor DEL‐22379 had no effects in their proliferative capacity (Fig. 1A), probably because they are not driven by RAS–ERK pathway oncogenes [17, 32]. However, when we evaluated their chemotactic response to EGF stimulation using Transwell assays, it was found that pretreatment either with the MEK inhibitor U0126, which completely blocked ERK activation, or with DEL‐22379, which specifically inhibited ERK dimerization without affecting ERK phosphorylation (Fig. 1B; Fig. S2A), markedly prevented cellular migration. This was also the case in cells transfected with the ERK2 HL mutant, which acts in a dominant inhibitory fashion preventing ERK dimerization [10] (Fig. 1C; Fig. S2B). These data indicated that the observed negative impact of the ERK dimerization inhibitor on cellular motility could not be attributed to a gross cytotoxic effect.

To analyze this observation in further detail we studied the effect of inhibiting ERK dimerization on different motility parameters in single cells. It was found that treatment with DEL‐22379 significantly diminished the total distance traveled by cells, both control and EGF‐treated, as evaluated by the mean square displacement (Fig. 1D), it also reduced their speed (Fig. 1E) and distorted their directionality, particularly in the case of EGF‐treated cells (Fig. 1F; Fig. S2C). Similarly, origin plots revealed a marked restriction on cell random movements following the administration of the dimerization inhibitor (Fig. 1G).

In order not to limit our analyses to a single cell line, we performed similar experiments on MDA‐MB‐231 cells, also of mammary tumor origin, though with a more prominent invasive potential [33]. As in the case of MCF7 cells, the proliferation rate of MDA‐MB‐231 cells was also unaffected by DEL‐22379 treatment (Fig. S3A), which, once again, did not affect ERK phosphorylation (Fig. S3B). Contrarily, EGF‐stimulated chemotaxis (Fig. S3C) and single cell total distance traveled (Fig. S3D) and velocity (Fig. S3E) were significantly diminished following treatment with the inhibitor. Using these cells, we also tested the effects of inhibiting ERK dimerization on three‐dimensional (3D) cellular movement, as observed in collagen‐embedded spheroids formed by these cells. In this setting, it was found that U0186 prevented spheroid growth. On the other hand, DEL‐22379 did not affect growth but evoked a pronounced reduction on the spontaneous dispersal from the MDA‐MB‐231 cellular masses exhibited by these cells as a consequence of their invasive behavior (Fig. S3F).

**Fig. 2:**
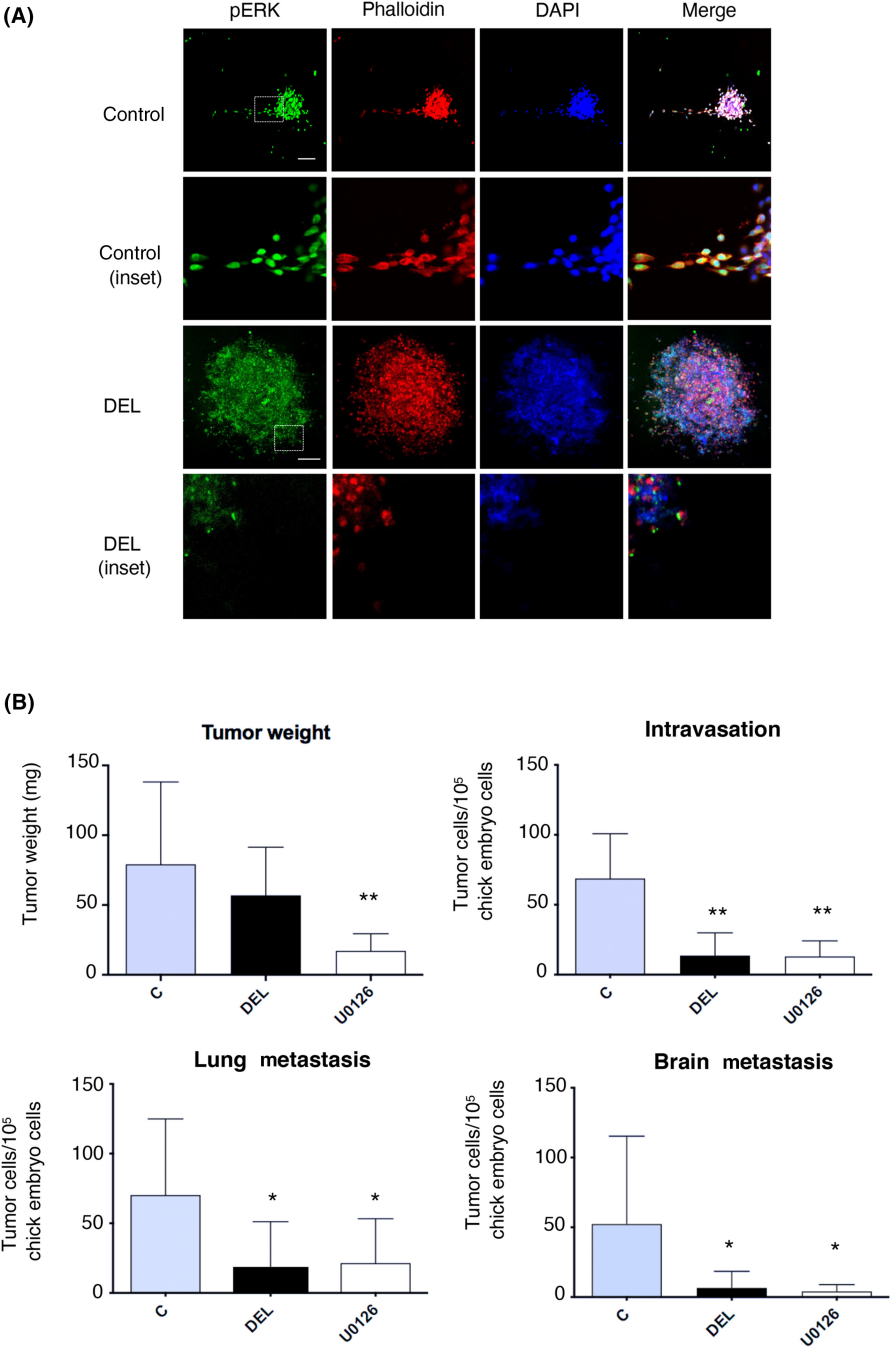
Inhibition of ERK dimerization impedes spontaneous metastases. (A) Inhibition of ERK dimerization prevents cells outspreading from MDA‐MB‐231 spheroids, as determined by immunofluorescence of spheroids treated with DEL‐22379 (10 μm, 1 h). Spheroids treated with DEL are shown at higher magnification to highlight the absence of evading cells. Scale bars: 50 μm. Results are representative of three independent experiments. (B) Effects of ERK dimerization inhibition on the indicated metastatic parameters in chick embryos whose CAM had been grafted with MDA‐MB‐231 and subsequently treated with DEL‐22379 or U0126 (each 10 μm/3 days). Data show mean ± SEM of three independent experiments. *P* values: **P < 0.01, *P < 0.05 by double‐tailed, unpaired Student *t*‐test.

Taking advantage of MDA‐MB‐231 cells spontaneous invasive capacity we investigated how the inhibition of ERK dimerization affected the metastatic potential of these cells. As mentioned before, treatment of the aforementioned MDA‐MB‐231 spheroids with DEL‐22379 resulted in pronounced attenuation on the outspreading of individual cells away from the main cellular mass (Fig. 2A). Accordingly, we also evaluated their *in vivo* metastatic potential in an animal model, by analyzing their ability to intravasate and to colonize distant organs using the chick chorio‐allantoid membrane model [17]. It was found that, unlike U0126, DEL‐22379 treatment did not overtly affect the primary tumor size. However, it markedly diminished the number of intravasated cells and of those invading the brain and the lung (Fig. 2B). Overall, these sets of data demonstrate that the inhibition of ERK dimerization prevents cell motility of mammary cells, thereby compromising their invasive potential.

### 3.2 ERK dimerization orchestrates actin architecture

**Fig. 3:**
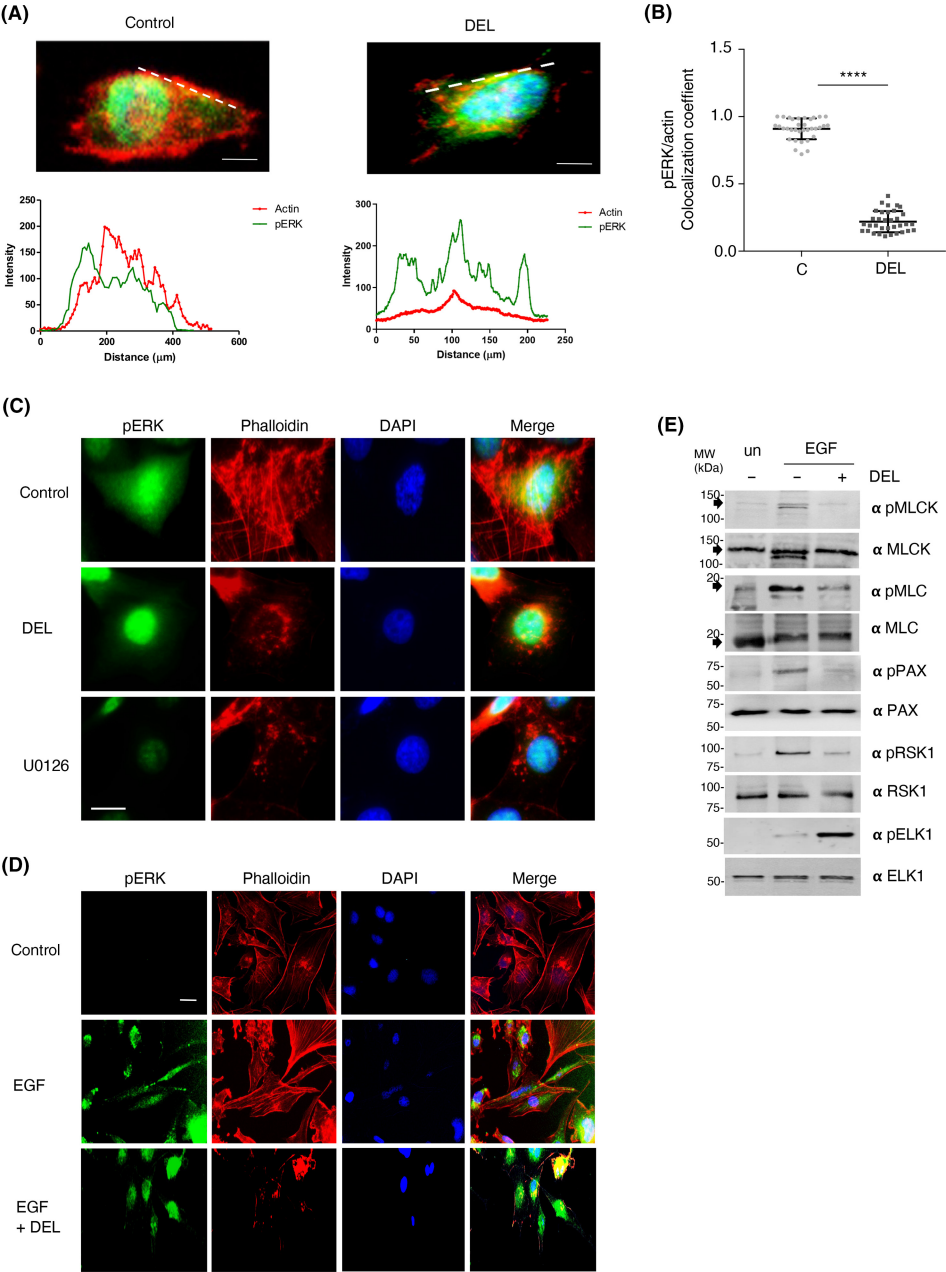
Inhibition of ERK dimerization affects actin cytoskeleton architecture. (A) Alterations on phosphorylated ERK colocalization with actin, evaluated by immunofluorescence micrographs of individual MDA‐MB‐231 cells treated with DEL‐22379 (10 μm, 1 h). Colocalization of phospho‐ERK (green) and actin (red, phalloidin staining) was evaluated along the depicted dashed lines. Scale bar: 50 μm. Representative cells of B. (B) As in A, quantification corresponding to *n* = 40 cells/condition. Data show mean ± SEM. *P* values: ****P < 0.001 by double‐tailed, unpaired Student *t*‐test. c, control. (C) Alterations on actin architecture and cell morphology. Immunofluorescence micrographs of MDA‐MB‐231 cells, control or treated with the indicated inhibitors. Scale bar: 10 μm. (D) As in C, in MCF7 cells stimulated where indicated with EGF (50 ng·mL^−1^, 10 min). Scale bar: 5 μm. (E) Effects on the molecular effectors that regulate cell motility. Immunoblotting for the phosphorylated and total forms of the indicated proteins in MCF7 cells unstimulated (un) or treated with EGF, pretreated for 1 h (+) or not (−) with DEL‐22379. Arrowheads indicate the referred proteins. (C–E) Results are representative of three independent experiments.

Since the dynamic remodeling of the actin cytoskeleton lies at the heart of cellular movement, it was of interest to investigate how the inhibition of ERK dimerization affected actin architecture. When analyzing MDA‐MB‐231 individual cells, it was found that in control cells there was a substantial colocalization of ERK with actin at the cell periphery, which was completely lost following the inhibition of ERK dimerization by DEL‐22379 treatment (Fig. 3A,B).

When appraising in further detail the architecture of the actin cytoskeleton, it was observed that normally proliferating MDA‐MB‐231 cells exhibited abundant stress fibers, plus lamellipodia and filopodia projections (Fig. 3C). Noticeably, impeding ERK dimerization by DEL‐22379 administration resulted in a total disruption of the stress fibers network and in filopodia disappearance, in addition to a marked subcellular redistribution of actin, which accumulated at the nuclear periphery forming clusters. This was also the case in cells treated with U0126 (Fig. 3C).

A similar effect was observed in MCF7 cells, in which EGF treatment evoked a profuse formation of stress fibers, lamellipodia, and filopodia. The formation of such structures was completely abrogated by treatment with DEL‐22379, which also caused the perinuclear accumulation of actin (Fig. 3D).

It was important to determine how the inhibition of ERK dimerization affected the molecular machinery that regulates actin dynamics and, subsequently, cell motility. Myosin Light Chain Kinase (MLCK) is a direct substrate of ERK [34]. At its turn, MLCK phosphorylates Myosin Light Chain (MLC), stimulating actomyosin contractility required for the extension of lamellipodiae at the cell leading edge [35, 36]. It was found that treatment with DEL‐22379 markedly reduced both MLCK and MLC phosphorylation levels (Fig. 3E). Similarly, administration of the ERK dimerization inhibitor also resulted in a down‐regulation of paxillin phosphorylation levels (Fig. 3E), a central event in focal adhesion turnover [37, 38]. In this respect, focal adhesions, as revealed by phospho‐paxillin immunofluorescence, were almost completely absent in DEL‐22379‐treated cells (Fig. S4). We also analyzed the phosphorylation of the ERK substrate p90 Ribosomal S6 kinase (RSK1), responsible for the inactivation of Myosin Phosphatase Regulatory subunit (MYPT1), fostering rear end retraction [39]. As in the previous cases, the inhibition of ERK dimerization by DEL‐22379 led to a pronounced attenuation on RSK1 phosphorylation (Fig. 3E). Conversely, the inhibition of ERK dimerization potentiated nuclear events such as the phosphorylation of ELK1, as we have previously demonstrated [17]. Overall, the above results indicate that ERK dimerization is a key factor in the regulation of actin organization, as an orchestrator of actomyosin contractility, both in protrusions extension and rear end retraction, and for the formation of focal adhesions.

### 3.3 Migration is specifically evoked by ERK‐stimulating agonists that induce its dimerization

**Fig. 4:**
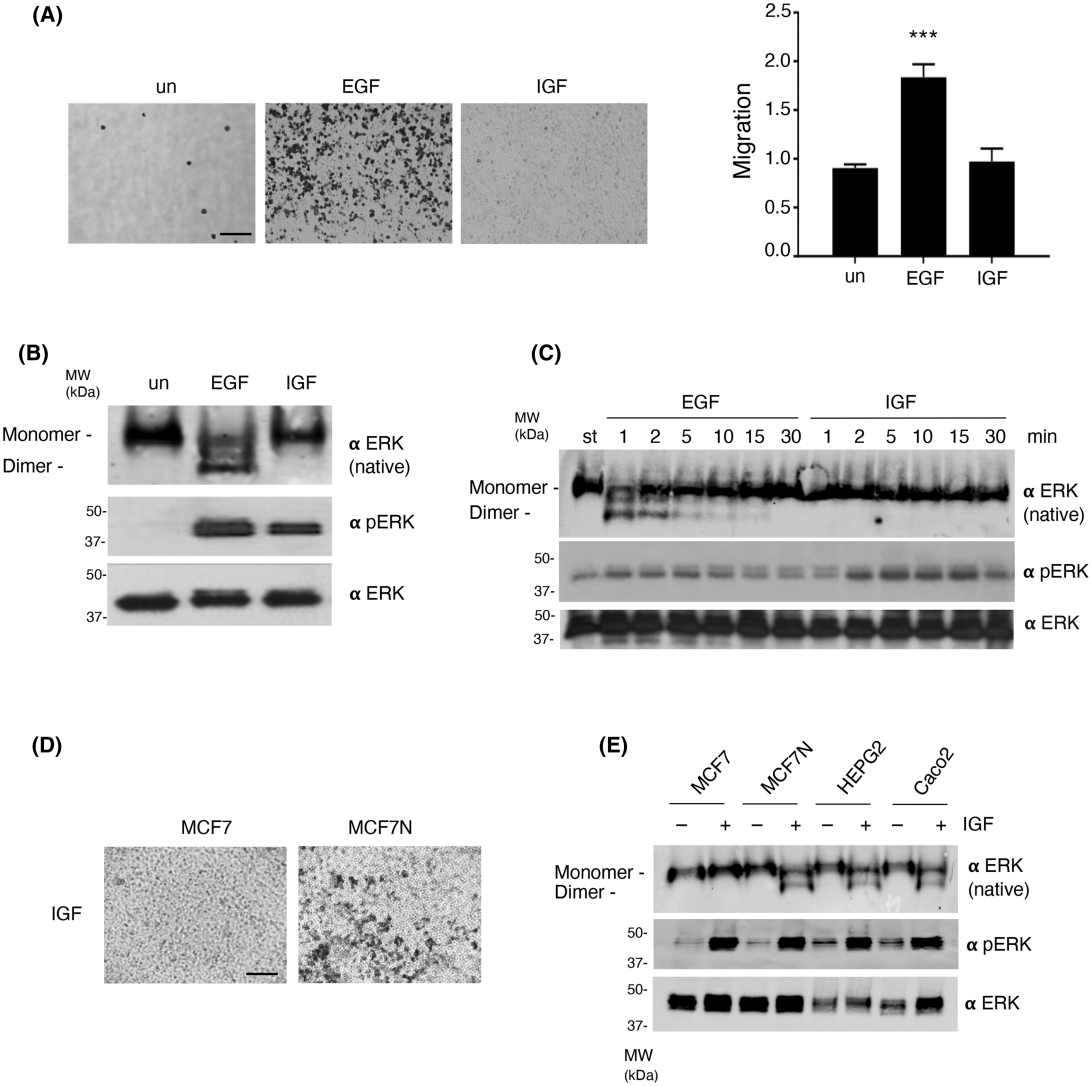
Cellular migration depends on the external agonists capacity for inducing ERK dimerization. (A) Left: Transwell cellular migration of MCF7 cells, unstimulated (un) and treated with EGF (50 ng·mL^−1^) or IGF‐1 (25 ng·mL^−1^) for 48 h, as chemoattractants. Scale bar: 50 μm Right: Quantification of three independent experiments. Data show mean ± SEM of three independent experiments. *P* values: ***P < 0.005 by double‐tailed, unpaired Student *t*‐test. (B) ERK dimerization and phosphorylation in MCF7 cells as induced after 5 min of stimulation with the indicated agonists. (C) Kinetics of ERK dimerization and phosphorylation in MCF7 cells as induced by treatments with the shown agonists for the indicated times. St, starved. (D) Transwell cellular migration of MCF7 and MCF7N cells, stimulated with IGF‐1 for 48 h. Scale bar: 50 μm. (E) ERK dimerization and phosphorylation as induced by IGF‐1 treatment (25 ng·mL^−1^, 5 min) in the indicated cell lines. (B–E) Results are representative of three independent experiments.

Not to restrict our quest to a single ERK‐activating, migration‐inducing stimulus, we also analyzed the effects on cell motility and ERK dimerization of Insulin‐like Growth Factor 1 (IGF‐1), previously reported to stimulate migration in MCF7 cells [40]. Surprisingly, this was not the case in our cells, as determined in Transwell assays using IGF‐1 as chemoattractant, as opposed to EGF (Fig. 4A). Noticeably, in our cells IGF‐1 was also incapable of eliciting ERK dimerization, in spite of readily inducing ERK phosphorylation (Fig. 4B), a phenomenon observed throughout an ample stimulation time range (Fig. 4C).

It was conceivable that our MCF7 cells had undergone some mutational event that made them insensitive to IGF‐1 migration‐stimulating effect. To test this possibility, we obtained an original batch of cells (MCF7N hereafter), and found that, unlike our MCF7 cells, in MCF7N cells IGF‐1 elicited a pronounced migratory response (Fig. 4D). In this respect, MCF7N migration could be stimulated also by EGF and in both cases migration could be inhibited by DEL‐22379 treatment (Fig. S5). In agreement, it was found that in MCF7N cells IGF‐1 stimulation produced a marked ERK dimerization, contrarily to our MCF7 batch (Fig. 2E). IGF‐1 also induced a potent ERK dimerization, in addition to ERK phosphorylation, in HEPG2 and Caco2 cells (Fig. 4E), cell types in which IGF‐1 has been previously shown to evoke cell motility [41, 42]. Overall, these results demonstrate that in order to stimulate cellular movement, growth factors signaling through the ERK pathway must be capable of inducing ERK dimerization, not solely ERK activation.

### 3.4 KSR1 couples growth factor signaling to ERK dimerization and cellular motility

It was of interest to understand why in the same cellular context ERK‐activating growth factors diverged in their ability to induce ERK dimerization and evoke cellular migration. Since Scaffold Proteins are known to be essential mediators in the ERK dimerization process, serving as dimerization platforms [7], we reasoned that the intervention of some specific scaffold could determine the capacity of an external agonist for stimulating ERK dimerization and, subsequently, cellular movement.

**Fig. 5:**
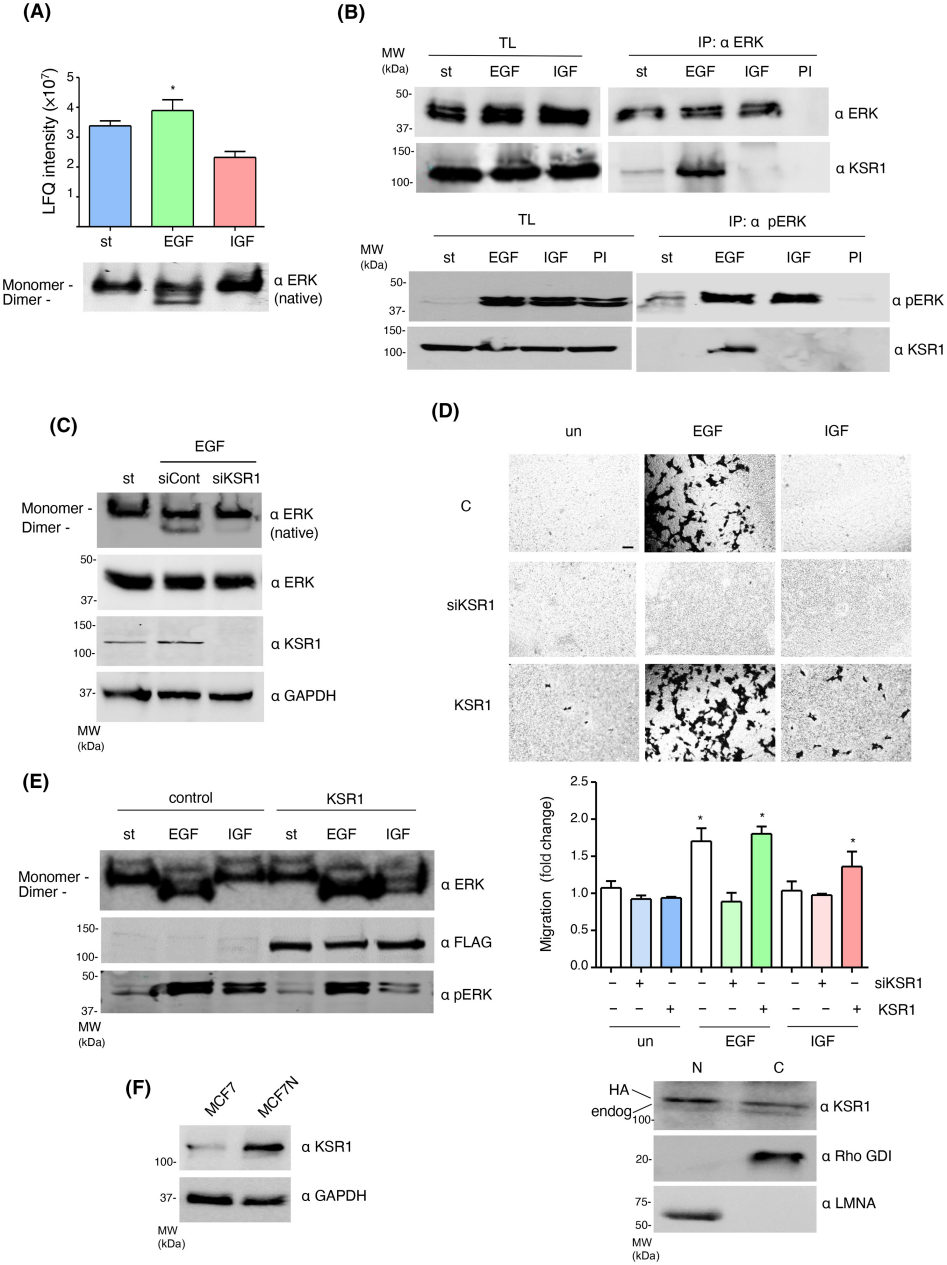
KSR1 regulates ERK dimerization and cell motility. (A) Mass spectrometry data showing the presence of KSR1 in anti‐HA immunoprecipitates from MCF7 cells starved (st) or stimulated with EGF (50 ng·mL^−1^) or IGF (25 ng·mL^−1^) for 5 min. Data represent average ± SEM LFQ (label‐free quantitation) intensity from three independent experiments. Lower panel: Induction of ERK dimerization in similar samples. (B) KSR1 interaction with ERK in MCF7 cells, starved (st) or treated with the indicated agonists for 5 min. Revealed by co‐immunoprecipitation, upon immunoprecipitation (IP) with the indicated proteins or with pre‐immune serum (PI), and subsequent western blotting. TL, total lysates. (C) Effect of KSR1 down‐regulation on ERK dimerization in EGF‐stimulated MCF7 cells transfected with control and anti‐KSR1 siRNAs where indicated. (D) Transwell cellular migration of MCF7 cells, parental (c) or transfected with an siRNA against KSR1 (siKSR1); or a vector encoding for KSR1 (1 μg each), without stimulation (un) or treated with the indicated agonists for 48 h. Scale bar: 50 μm. Middle panel: Data show mean ± SEM relative to the values of parental, unstimulated cells, from three independent experiments. *P* values: *P < 0.05 by double‐tailed, unpaired Student *t*‐test. Lower panel: KSR1 levels and nucleo‐cytoplasmic distribution in the KSR1‐trasfected cells. The endogenous and HA‐tagged, ectopic KSR1 bands are indicated. Rho‐GDI and LaminA mark the cytoplasmic (C) and nuclear (N) fractions respectively. (E) Effect of KSR1 overexpression on ERK dimerization in EGF‐stimulated MCF7 cells in response to stimulation with the indicated agonists for 5 min. (F) KSR1 expression levels in the indicated cell lines. (B–F) Results are representative of three independent experiments.

To test this hypothesis, we performed an unbiassed search for scaffold protein species which associated to phosphorylated ERK2 in response to EGF and IGF‐1 stimulation in MCF7 cells, using mass spectrometry. Our analyses revealed a significant binding of Kinase Suppressor of RAS 1 (KSR1) to activated ERK in EGF but not in IGF‐1 treated cells (Fig. 5A). This finding was ascertained by co‐immunoprecipitation assays, which demonstrated that the association between ERK and KSR1 was substantially increased in response to EGF, as opposed to IGF‐1 stimulation (Fig. 5B). Next, we investigated the relevance for ERK dimerization of the interaction occurring between ERK and KSR1. To do so, we depleted MCF7 cells of KSR1 by means of an siRNA, and found that, indeed, down‐regulation of KSR1 levels markedly reduced EGF competence for inducing ERK dimerization (Fig. 5C). In agreement, when we tested the effects of altering KSR1 levels on cell motility, it was found that depletion of KSR1 completely prevented EGF‐stimulated cellular migration (Fig. 5D). On the other hand, the overexpression of KSR1 did not affect EGF high capacity for inducing cell motility, but it endowed IGF‐1 with the faculty for promoting a significant extent of cellular migration (Fig. 5D). In consonance, KSR1 overexpression was found to restore IGF‐1 ability for evoking a substantial amount of ERK dimerization (Fig. 5E). In full conformity, MCF7N cells, where IGF‐1 induces both ERK dimerization and migration, displayed much higher KSR1 levels than MCF7 cells, which don't respond to IGF‐1 (Fig. 5F). Taken together, these sets of data demonstrate that KSR1 plays a central role in the connection of ERK‐activating signals induced by extracellular agonists to ERK dimerization and the regulation of cellular motility.

### 3.5 KSR1 regulates the actin cytoskeleton and cellular morphology

**Fig. 6:**
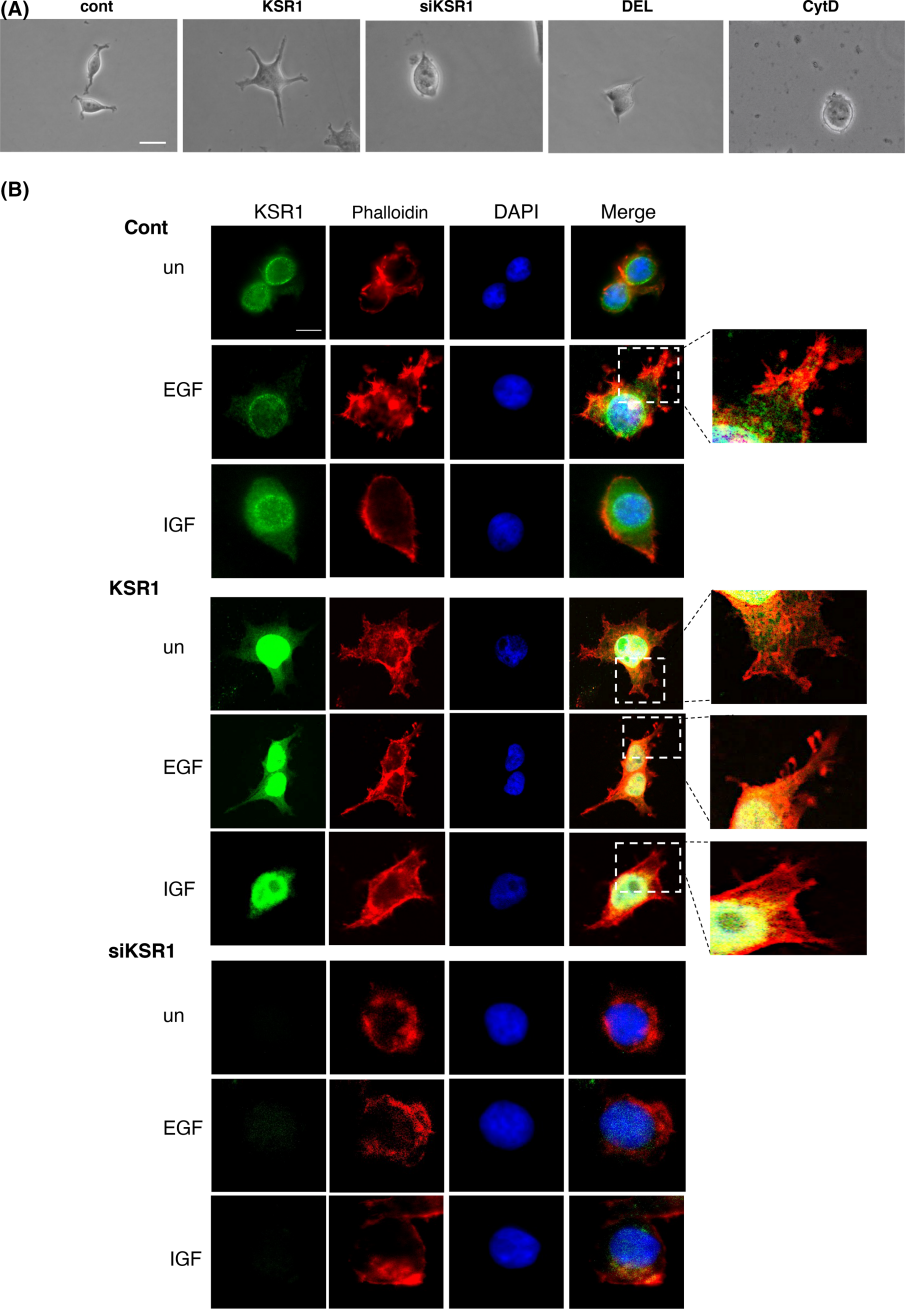
KSR1 regulates the actin cytoskeleton and cellular morphology. (A) Phase contrast micrographs showing the representative morphology of individual MCF7 cells, parental (control); overexpressing KSR1; siRNA‐mediated, downregulated KSR1 levels (siKSR) and treated with DEL‐22379 or cytochalasin D (10 μm, 1 h each). Scale bar: 10 μm. (B) Alterations on actin architecture and cell morphology, in MCF7 cells: parental (cont); over‐ (KSR1) or under‐expressing KSR1 (siKSR1), when unstimulated (un) or treated with EGF (50 ng·mL^−1^) or IGF (25 ng·mL^−1^) for 1 h. KSR1 (green) and actin (phalloidin, red) distributions are shown. Scale bars: 10 μm. Inset magnifications reveal lamellipodia and filopodia formations. (A, B) Results are representative of three independent experiments.

It was probable that the changes on cell motility in response to agonists stimulation, as a consequence of KSR1 over‐ or under‐expression, stemmed from its impact on the actin architecture. In this respect, we observed that the sole alteration of KSR1 levels clearly affected cellular morphology. KSR1 overexpression markedly altered the usual spindle shape of MCF7 cells, ensuing flattened cells enriched in membrane protrusions, particularly filopodia. Conversely, KSR1 depletion resulted in rounder cells with less protrusions, an appearance resembling that of cells treated with DEL‐22379, or that generated by the actin cytoskeleton‐disrupting drug cytochalasin D (Fig. 6A; Fig. S6A).

When we analyzed if alterations on KSR1 levels affected the actin cytoskeleton in response to growth factor stimulation, it was found that KSR1 overexpression did not substantially affect the morphology of EGF‐treated cells, characterized by profuse lamellipodia and filopodia extensions. Conversely, it markedly altered the rounded appearance of control and IGF‐1‐treated cells to a more “EGF‐like” flattened morphology, displaying lamellipodia and filopodia, the latter more prominent in cells stimulated with IGF‐1 (Fig. 6B, insets; Fig. S6B). Of note, KSR1 at physiological levels exhibited a uniform nucleo‐cytoplasmic distribution accumulating around the nucleus, whereas overexpression resulted in a marked nuclear enrichment though also overexpressed at the cytoplasm, as previously reported [43] (Fig. 5D, lower panel). On the other hand, in all cases KSR1 depletion resulted in rounded cells with actin accumulating at the nuclear periphery (Fig. 6B), resembling those treated with the ERK dimerization inhibitor (Fig. 3B; Fig. S6B). Overall, these results demonstrate that KSR1 plays a determinant role in the regulation of the actin cytoskeleton and, as a consequence, on cellular morphology.

### 3.6 KSR1 levels determine the clinical outcome of mammary tumors

**Fig. 7:**
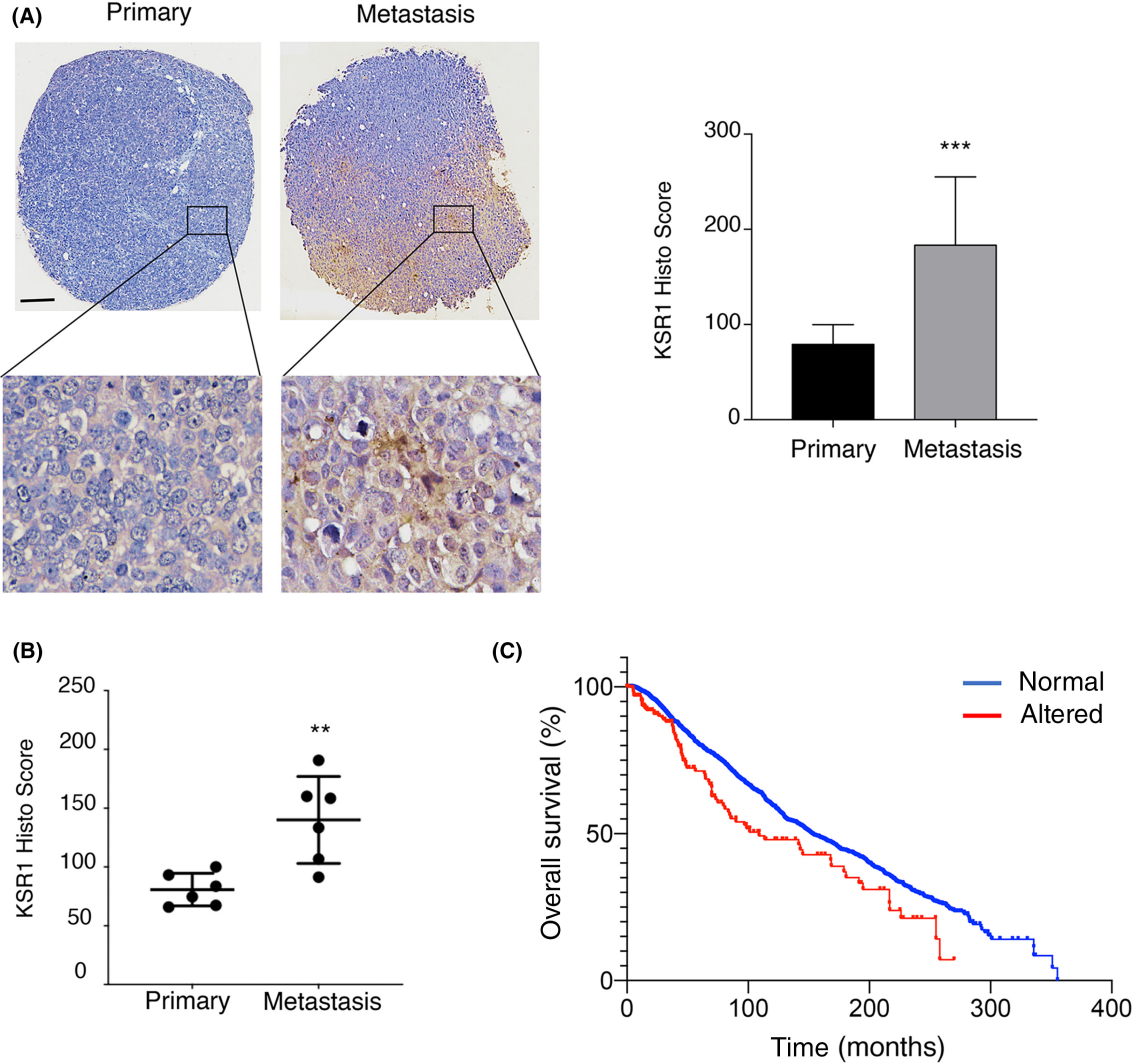
KSR1 alterations in clinical samples. (A) Expression of KSR‐1 in primary tumor and metastasis breast cancer samples. Left panel: Representative thin sections of formalin‐fixed paraffin‐embedded tissues from primary and metastatic tissues from a broad‐spectrum breast disease TMA, stained with antibody against KSR‐1 Scale bar: 200 μm. Right panel: *H*‐score of KSR1 quantification corresponding to 51 patients, displayed as DAB signals in 20× field, 8 fields per slice using color deconvolution TMA plugin fiji software. Results are representative of three independent experiments. (B) As in A, quantification pertaining to samples coming from the same patient (*n* = 6). (A, B) Data show average ± SEM. *P* values: **P < 0.05; ***P < 0.01 by double‐tailed, unpaired Student *t*‐test. (C) Kaplan–Meier survival curves after diagnosis (time) corresponding to 6344 breast cancer patients with normal vs altered KSR1 expression levels.

Since our data implicated KSR1 in the regulation of cellular motility of mammary cells, we sought to gain an insight into the clinical relevance of this finding, by analyzing KSR1 levels in a TMA made up of 51 PDXs coming both from primary mammary tumors and metastases. Remarkably, it was found that KSR1 levels were significantly higher in samples corresponding to metastases, compared to primary tumors in general (Fig. 7A). This was also the case, when comparing KSR1 levels in the primary tumor versus metastasis in samples coming from the same patient (Fig. 7B). Following these results, we searched the available databases in order to analyze in further depth how KSR1 levels influenced the clinical evolution of breast cancer patients. Data corresponding to 6344 breast cancer cases included in five different repositories, revealed that 4% of these cases exhibited genetic alterations in the KSR1 locus, amplifications almost in every case, which, conceivably, should result in augmented KSR1 levels (Fig. S7). Remarkably, there was a significant reduction on the survival rate of the patients harboring KSR1 alterations compared to those with a normal KSR1 genotype (Fig. 7C), in full agreement with our results demonstrating the relevance of KSR1 for the promotion of cell motility and invasion.

### 3.7 ERK dimerization is sufficient for promoting cell motility

**Fig. 8:**
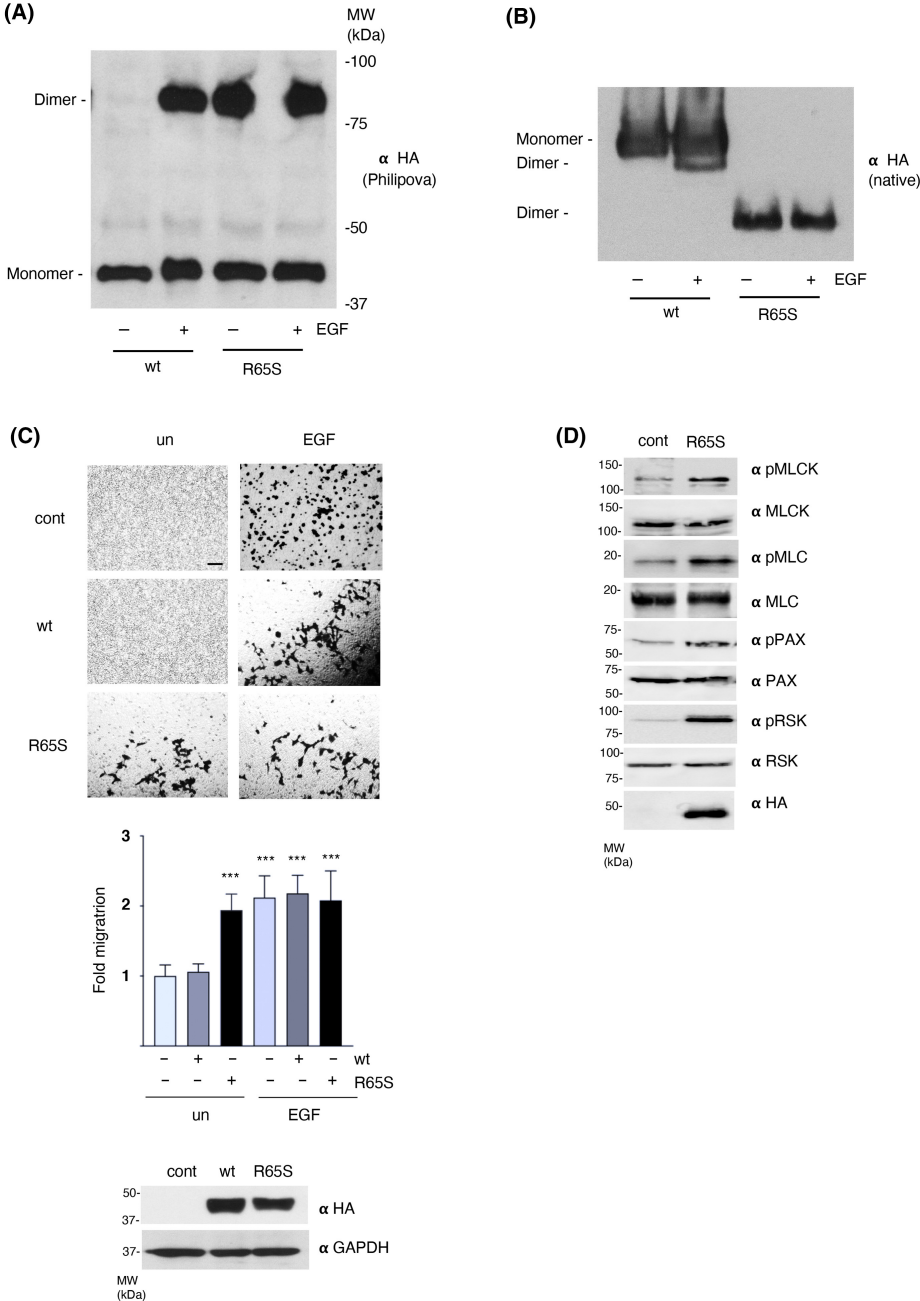
Constitutive ERK dimerization drives cell motility. (A) ERK2 R65S is in a constitutively dimeric state. As revealed by “Philipova” PAGE of cell extracts from cells transfected with HA‐tagged ERK2, wild‐type (wt) or R65S (1 μg each), starved (−) or stimulated (+) with EGF (50 ng·mL^−1^, 5 min). (B) As in A, resolved in native PAGE. (C) Transwell cellular migration of MCF7 cells, parental (cont) or transfected with ERK2 wt or R65S (1 μg each), unstimulated (un) or treated with EGF for 48 h. Scale bar: 50 μm. Middle panel: Data show mean ± SEM relative to the values of parental, starved cells, from three independent experiments. *P* values: ***P < 0.001 by double‐tailed, unpaired Student *t*‐test. Lower panel: expression levels of the transfected HA‐tagged ERK2 wt and R65S. (D) Effects of ERK2 R65S on the molecular effectors that regulate cell motility. Immunoblotting for the indicated phosphorylated and total forms of the indicated proteins in MCF7 cells: parental (cont) or transfected with HA‐tagged ERK2 R65S. (A–D) Results are representative of three independent experiments.

In light of our previous evidence demonstrating that ERK dimerization is an absolute requisite for cellular movement to take place, we asked whether it could also suffice to drive cellular motion. To this end, we used the ERK2 intrinsically active mutant R65S [44]. In addition to being hyperactive regarding its kinase activity [44], we observed that this mutant is constitutively in dimeric form even in unstimulated conditions, as demonstrated by the “Philipova” SDS/PAGE method [5, 7] (Fig. 8A) and by native electrophoresis (Fig. 8B). Of note, the latter technique revealed that ERK2 R65S dimers have a higher electrophoretic mobility than wild‐type dimers, probably because the arginine to serine substitution decreases their positive charge.

Even though ERK2 R65S is uncapable of driving cellular transformation and proliferation by itself [45], it was found that it could effectively drive cellular migration, even under unstimulated conditions when transfected in MCF7 cells (Fig. 8C). In agreement with this observation, we found that cells expressing ERK2 R65S exhibited augmented levels of phosphorylated MLCK, MLC, paxillin and RSK (Fig. 8D), characteristic of moving cells. Overall, these results demonstrate that ERK dimerization is sufficient for supporting cell motility.

## 4 Discussion

While dimerization in response to stimulation is a widespread phenomenon in the RAS–ERK pathway [46] its significance remains obscure. This is particularly true in the case of ERK. Its dimerization has been described to be an important factor for its spatial regulation [7, 10] and for carcinogenesis [17]. However, its implications in specific cellular events remain obscure. Here, we unveil its utmost importance for cell motility, a process in which ERK participation has been widely reported [1, 23, 24], in particular in mammary tumor cells, where ERK activation seems to play an important role in their dissemination [25].

Our data indicating that cell motility is severely compromised both by the inhibition of ERK dimerization and by the drop on the levels of scaffold proteins like KSR1, strongly suggest that the regulation of cell motility occurs mainly at the cytoplasm, the cellular compartment where ERK dimers operate [7, 17] and scaffold proteins activity primarily unfolds [7, 10, 13]. This is in agreement with previous findings, showing that inhibiting ERK cytoplasmic substrates like RSK1 prevents cytoskeletal rearrangements necessary for motility, within minutes and independently of transcriptional events [39, 47]. And with the fact that ERK plays a major regulatory role in the dynamics of the cytoskeletal architecture, at the cytoplasm, required for each and every step of the cellular movement process involving the actin framework [24], as we demonstrate herein.

Our results also demonstrate that growth factors acting through tyrosine kinase receptors, perfectly competent for evoking ERK phosphorylation/activation, must be capable of also inducing ERK dimerization in order to unleash cellular movement. In this respect, our data showing that the intrinsically active [44] and constitutively dimeric ERK2 R65S mutant, can promote cell motility by itself, clearly indicates that in human mammary cells ERK dimers must be undertaking tasks that activated ERK monomers cannot accomplish.

In this respect, we unveil the critical role played by the scaffold protein KSR1 in coupling RTK's to ERK dimerization and the unleashing of cellular motion. We demonstrate that a specific RTK agonist, in this case IGF‐1, always competent for inducing ERK phosphorylation/activation, will evoke ERK dimerization and trigger cell motility only if KSR1 is expressed at optimal levels. Remarkably, another growth factor, EGF, is less demanding when fostering KSR1‐dependent ERK dimerization and, subsequently, cell motility. While the mechanism underlying such differential affinity remains unknown, one possibility could be that KSR1 is enriched in those membrane microdomains where EGF receptors reside, whereas higher KSR1 levels would be necessary to stock those locations where IGF‐1 receptors are present. Be as it may, our data suggests that different stimuli display distinct capabilities for recruiting KSR1 to participate on ERK dimerization, and point to KSR1, among other types of scaffold proteins, as a critical factor in the ERK dimers assembly process.

In consonance, our results point to KSR1 as a critical orchestrator of cellular migration, in full agreement with previous findings which implicate KSR1 in the regulation of the actin cytoskeleton [48, 49] and, consequently, in the control of cell motility with pathological connotations in tumor cells [50]. Specifically pertinent to mammary tumor cells, dealt with herein, it has been demonstrated that the anti‐metastatic protein Nm23‐H1 precludes cellular migration [51]. Noticeably, cells expressing high levels of Nm23‐H1 exhibit a pronounced KSR1 degradation [52], suggestive of a correlation between KSR1 levels and an adverse evolution of mammary cancer patients, as our findings related to clinical data demonstrate.

Drugs targeting RAS–ERK pathway constituents are not habitual first‐line options for the treatment of mammary malignancies. Mainly because these tumors are rarely driven by RAS–ERK pathway oncogenes, therefore they are largely insensitive to these drugs, and more efficient therapeutic alternatives are available [53]. However, our findings showing the importance of ERK dimerization for the motility and metastatic dissemination of mammary tumor cells, open an interesting venue for the future development of ERK dimerization inhibitors, either by directly targeting ERK–ERK interaction [17] or ERK‐KSR1 association [2]. Such inhibitors could prove useful, in combination with current therapeutics, to prevent the metastatic spreading of mammary tumors.

Finally, it is worth noticing that our results disclosing an essential role of ERK dimerization in cell motility, pertain to the mammalian cell. Remarkably, ERK dimerization is a phenomenon that only takes place in mammalians, not in other organisms [17]. As cells in other creatures also undergo migratory events, it follows that in these ERK must regulate cellular motion as a monomer. The evolutionary advantage provided to mammalians by ERK acting in dimeric form in the regulation of migration, a critical process for development and organismal homeostasis, remains to be established.

## 5 Conclusions

Our results demonstrate that ERK dimerization, not solely its activation, is an essential factor for the regulation of cell motility and mammary tumor dissemination. In this process, the scaffold protein KSR1 is a critical element for enabling external agonists to induce ERK dimerization and, subsequently, to unleash cellular motion.

## Conflict of interest

The authors declare no conflict of interest.

## Author contributions

PC and BC contributed to conceptualization, supervision, and revision. PC, BC, AK, JA, MG, and JL contributed to methodology. DF‐V, RG‐G, JR, VC, BC, SD‐S, SV‐D, and CA contributed to investigation. BC contributed to validation. BC and RG‐G contributed to formal analysis. PC contributed to writing—original draft and writing—review and editing. DF‐V and BC contributed to visualization. PC, BC, AK, and JA contributed to funding acquisition. PC, BC, JA, and JL contributed to resources.

### Peer review

The peer review history for this article is available at https://www.webofscience.com/api/gateway/wos/peer‐review/10.1002/1878‐0261.13732.

## Supporting information


**Fig. S1.** Mycoplasma tests for the cell lines utilized.
**Fig. S2.** Inhibition of ERK dimerization prevents cell motility in MCF7 cells.
**Fig. S3.** Inhibition of ERK dimerization prevents cell motility in MDA‐MB‐231 cells.
**Fig. S4.** Inhibition of ERK dimerization affects focal adhesions formation.
**Fig. S5.** Cell motility of MCF7N cells in response to agonists and inhibition of ERK dimerization.
**Fig. S6.** Alterations on cell morphology of MCF7 cells as a consequence of alterations on KSR1 levels.
**Fig. S7.** KSR1 alterations on clinical samples.

## Data Availability

No data has been deposited. All reagents described herein are freely available upon request.
