# Supplementary material for: ERK1/2 mitogen‐activated protein kinase dimerization is essential for the regulation of cell motility
Source: Mol Oncol. 2024 Sep 12;19(2):452–73. doi: 10.1002/1878-0261.13732 (PMC11792999; doi:10.1002/1878-0261.13732)
Supplement: Supplementary file 1 — Fig. S1. Mycoplasma tests for the cell lines utilized. Fig. S2. Inhibition of ERK dimerization prevents cell motility in MCF7 cells. Fig. S3. Inhibition of ERK dimerization prevents cell motility in MDA‐MB‐231 cells. Fig. S4. Inhibition of ERK dimerization affects focal adhesions formation. Fig. S5. Cell motility of MCF7N cells in response to agonists and inhibition of ERK dimerization. Fig. S6. Alterations on cell morphology of MCF7 cells as a consequence of alterations on KSR1 levels. Fig. S7. KSR1 alterations on clinical samples. [file MOL2-19-452-s001.pdf]

Supp Figure 1

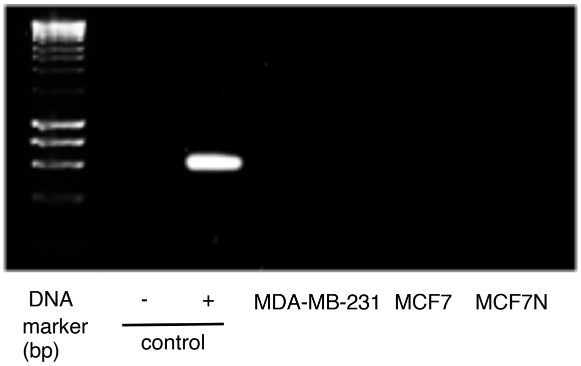

Supplemental Figure 1. Mycoplasma tests for the cell lines utilized.

Supp Figure 2

A

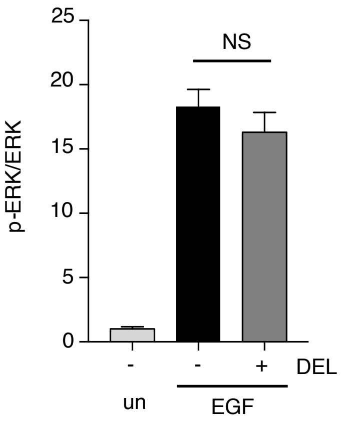

B

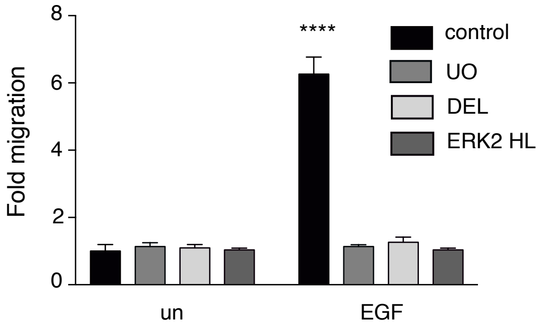

C

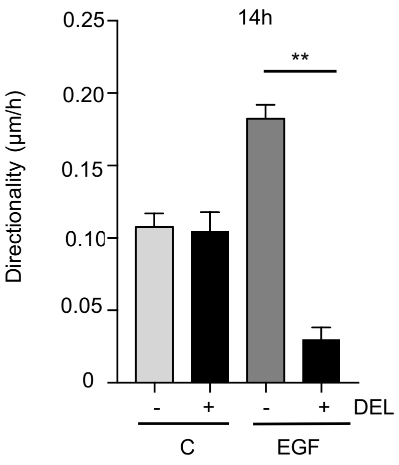

Supplemental Figure 2. Inhibition of ERK dimerization prevents cell motility in MCF7 cells. **A)** Effects of DEL-22379 on ERK phosphorylation. Quantifications corresponding to the western blot shown in Figure 1B plus two similar ones, showing the phosphorylated / total ERK ratio. Data shows average ± SEM from three independent experiments. P values: NS $p>0.05$ by double-tailed, unpaired Student t-test. **B)** Effects of ERK dimerization inhibition on cellular migration. Assayed in Transwell chambers (8 µm pore) using EGF (50 ng/ml, 48 h) as chemoattractant, in the presence of DEL-223379, U0126 or previously transfected with the ERK2 HL mutant (1 µg), where indicated. Data shows average ± SEM of three independent experiments relative to the levels found in unstimulated, control cells. P values: **** $p<0.001$ by double-tailed, unpaired Student t-test. **C)** Effects of ERK dimerization inhibition on cellular directionality. Detail corresponding to the 14h time point in Figure 1F. Data shows mean ± SEM of three independent experiments. P values: ** $p<0.01$ by double-tailed, unpaired Student t-test.

Supp Figure 3

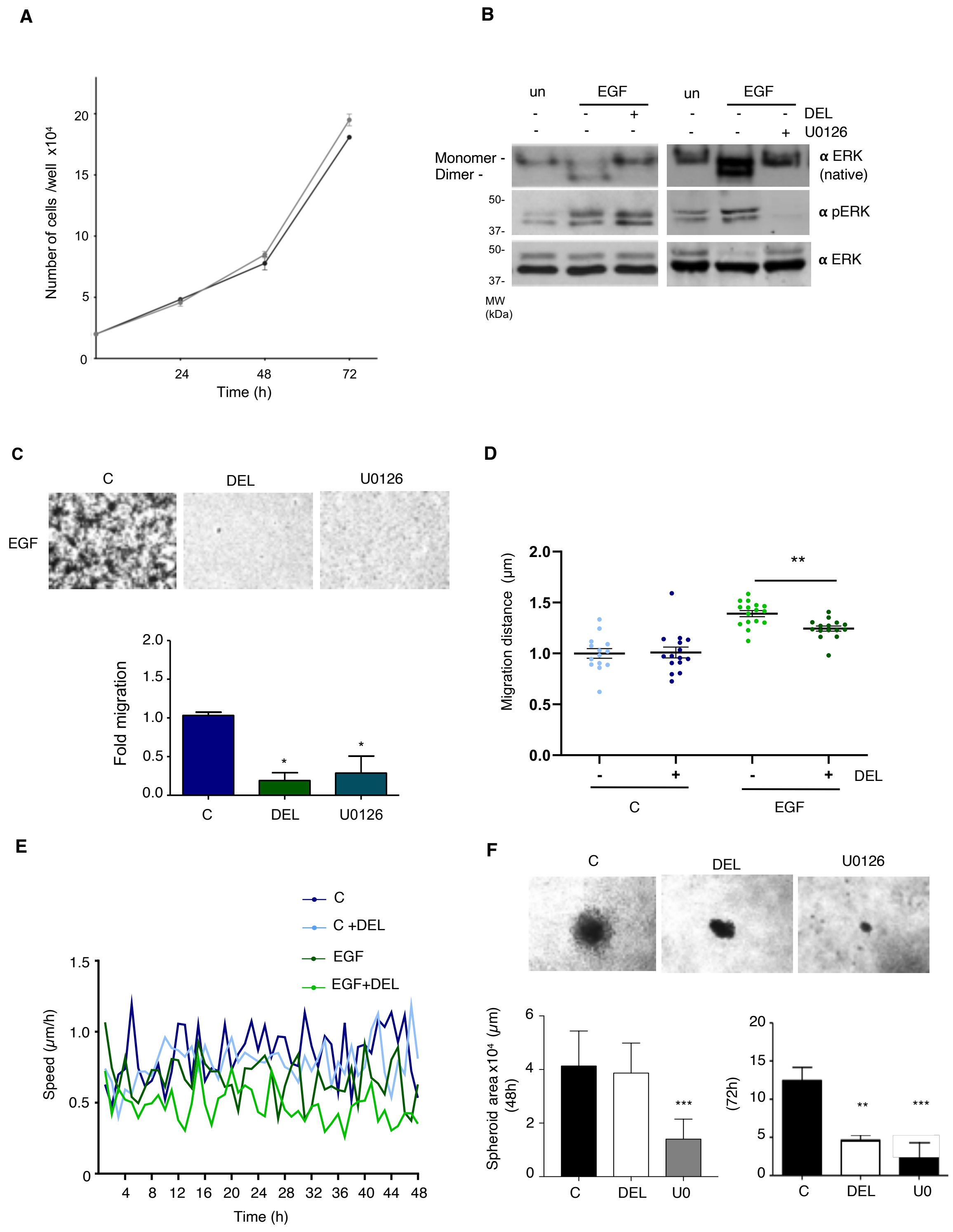

Supplemental Figure 3. Inhibition of ERK dimerization prevents cell motility in MDA-MB-231 cells. A) Effects of an ERK dimerization inhibitor on the proliferation of MDA-MB-231 cells. Where shown, cells were treated with DEL-22379 (10 μM) for the indicated times. **B)** Effects of DEL-22379 or U0126 (10 μM each) (+) on ERK dimerization and phosphorylation. Administered to 18h-starved cells 30 min before treatment with EGF (50 ng/ml, 5 min) where indicated. un= unstimulated cells. **C)** Effects of ERK dimerization inhibition on cellular migration. Assayed in Transwell chambers (8 μm pore) in the presence of DEL-223379 or U0126 (10 μM, 48h) using EGF (50 ng/ml) as chemoattractant. Data shows average ± SEM of three independent experiments. P values: * p<0.05 by double-tailed, unpaired Student t-test. **D E)** Effects of ERK dimerization inhibition on the indicated parameters of cell motility in control (C) or EGF-treated cells (50 ng/ml, 48 h), as indicated in each figure. Data shows average± SEM from three independent experiments. P values: ** p<0.01 by double-tailed, unpaired Student t-test. **F)** Inhibition of ERK dimerization prevents cells outspreading from MDA-MB-231 spheroids. As determined by phase contrast microscopy of spheroids, control (C) or treated with DEL-22379 or U0126 (10 μM) after 72h. Left graph: Spheroid area after 48h in the presence of the inhibitors. Right graph: Spheroid area after 72h in the presence of the inhibitors. Data shows mean ± SEM of three independent experiments. P values: ** p<0.01, *** p<0.005 by double-tailed, unpaired Student t-test.

Supp Figure 4

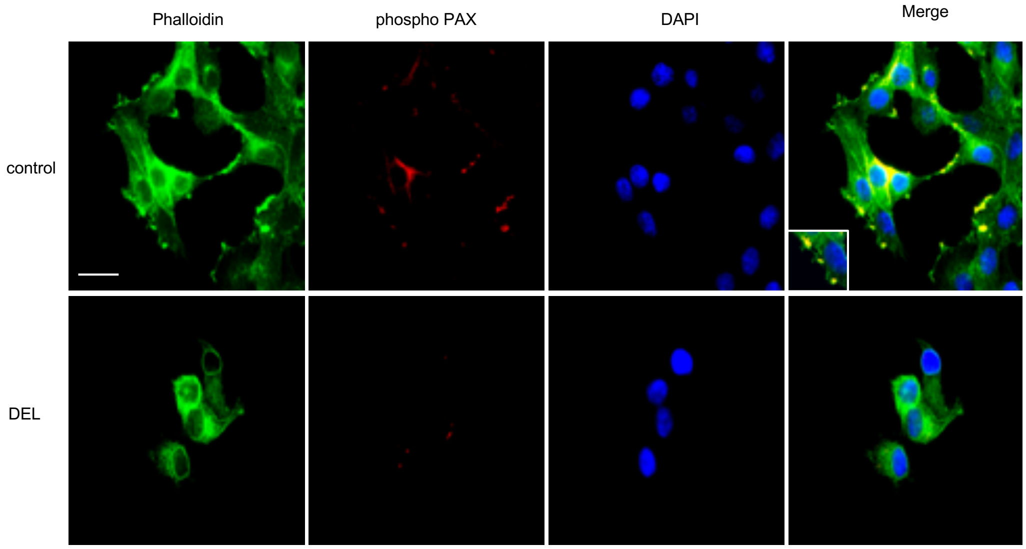

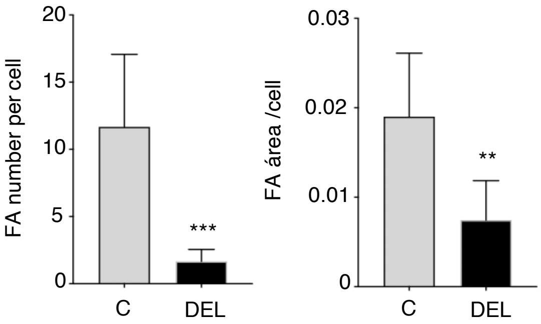

Supplemental Figure 4. Inhibition of ERK dimerization affects focal adhesions formation. Immunofluoresce micrographs of MDA-MB-231 cells growing (control) or treated with DEL-22379 (10 μM, 1h) showing the colocalization of actin (phalloidin, green) and phosphorylated paxillin (red). Inset shows accumulation of focal adhesions (yellow). Scale bar: 50 μm. Lower panels: quantifications corresponding to focal adhesions (FA) number and area per cell. Data shows mean ± SEM of 15 fields from three independent experiments P values: * $p<0.05$; ** $p<0.01$ by double-tailed, unpaired Student t-test.

Supp Figure 5

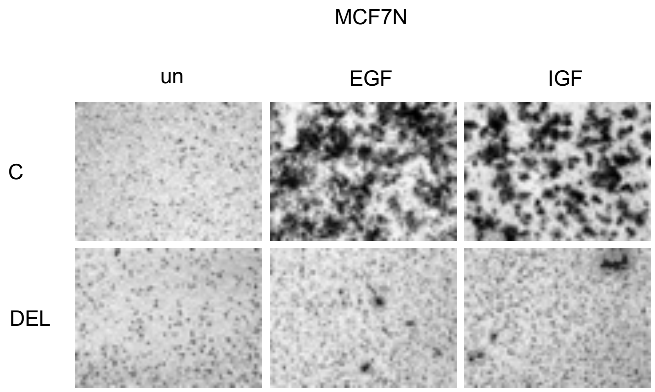

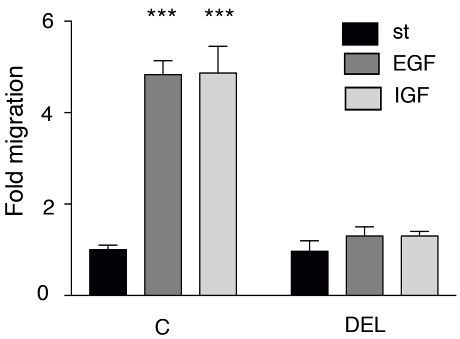

Supplemental Figure 5. Cell motility of MCF7N cells in response to agonists and inhibition of ERK dimerization. Assayed in Transwell chambers (8 μm pore) in the presence of DEL-223379 (10 μM) where indicated, using EGF (50 ng/ml) or IGF-1 (25 ng/ml) for 48h as chemoattractants. Data shows average ± SEM of three independent experiments relative to the values of control, unstimulated (un) cells. P values: *** $p<0.005$ by double-tailed, unpaired Student t-test.

Supp Figure 6

A

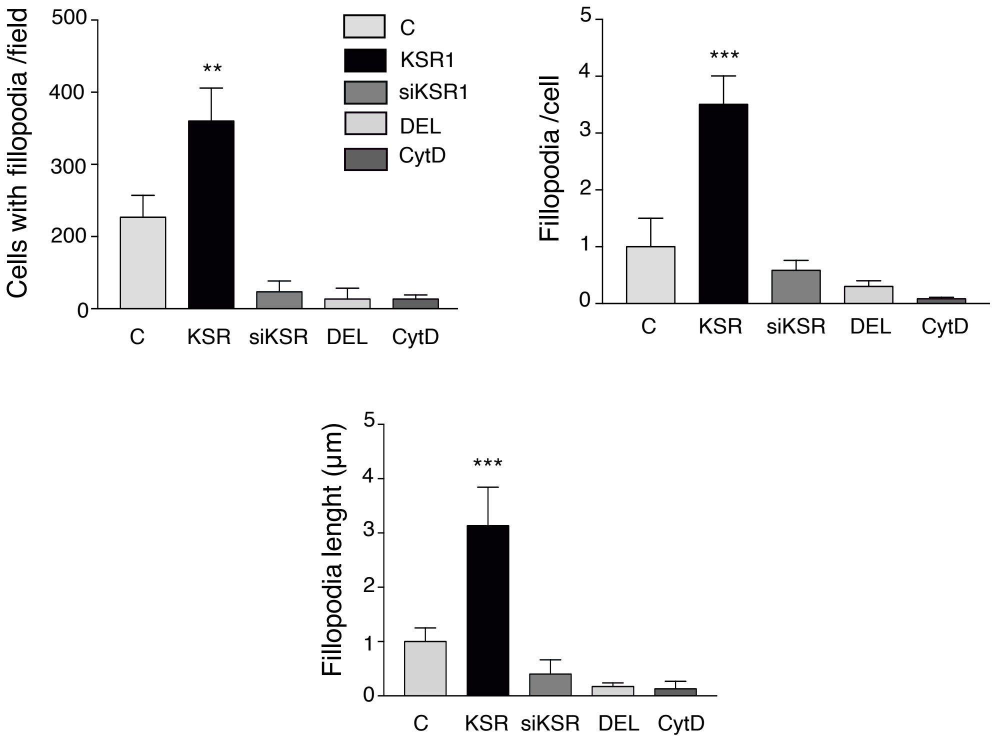

B

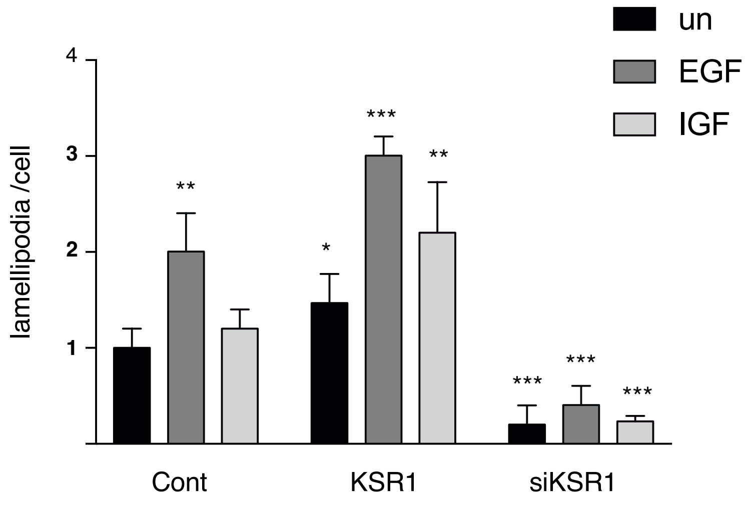

Supplemental Figure 6. Alterations on cell morphology of MCF7 cells as a consequence of alterations on KSR1 levels. A) Quantifications corresponding to Fig 6A, evaluating the: number of cells with filopodia; number of filopodia per cell (relative to those found in parental cells); and filopodia length (relative to the average length found in parental cells), in MCF7 cells, parental (c); overexpressing KSR1; siRNA-mediated, downregulated KSR1 levels (siKSR); and treated with DEL-22379 or cytochalasin D (10 μM, 1h each). Data shows average ± SEM of 12 fields from three independent experiments. P values: ** $p<0.01$; *** $p<0.005$ by double-tailed, unpaired Student t-test. **B)** Quantifications corresponding to Fig 6B, evaluating the number of lamellipodia per cell relative to those found in control, unstimulated cells, in MCF7 cells: parental (cont); over- (KSR1) or under-expressing KSR1 (siKSR1), when unstimulated (un) or treated with EGF (50 ng/ml) or IGF (25 ng/ml) for 1h. Data shows average ± SEM of 15 fields from three independent experiments. P values: ** $p<0.01$; *** $p<0.005$ by double-tailed, unpaired Student t-test.

Supp Figure 7

Data used in the analysis (n=6344):

· Breast Cancer (METABRIC, Nature 2021 & Nat Commun 2016): 2509 Samples
· Breast Invasive Carcinoma (TCGA, Cell 2015): 818 Samples
· Breast Invasive Carcinoma (TCGA, Firehose Legacy): 1108 Samples
· Breast Invasive Carcinoma (TCGA, Nature 2012): 825 Samples
· Breast Invasive Carcinoma (TCGA, PanCancer Atlas): 1084 Samples

Oncoprint

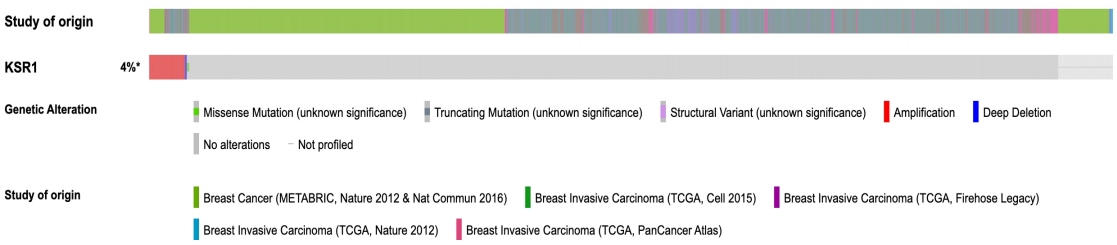

Statistical analysis:

Comparison of Survival Curves		
Log-rank (Mantel-Cox) test		
Chi square	11.24	
df	1	
P value	0.0008	
P value summary	***	
Are the survival curves sig different?	Yes	
Gehan-Breslow-Wilcoxon test		
Chi square	15.32	
df	1	
P value	<0.0001	
P value summary	****	
Are the survival curves sig different?	Yes	
Median survival		
Altered	109.0	
Unaltered	152.1	
Ratio (and its reciprocal)	0.7168	1.395
95% CI of ratio	0.5733 to 0.8961	1.116 to 1.744
Hazard Ratio (Mantel-Haenszel)	A/B	B/A
Ratio (and its reciprocal)	1.576	0.6345
95% CI of ratio	1.208 to 2.056	0.4863 to 0.8278
Hazard Ratio (logrank)	A/B	B/A
Ratio (and its reciprocal)	1.462	0.6842
95% CI of ratio	1.120 to 1.907	0.5245 to 0.8925

Supplemental Figure 7. KSR1 alterations on clinical samples. Database origin, Oncoprint and statistical evaluation of the 6344 breast cancer cases utilized in the analyses.
